# Rapid diversification of *homothorax* expression patterns after gene duplication in spiders

**DOI:** 10.1186/s12862-017-1013-0

**Published:** 2017-07-14

**Authors:** Natascha Turetzek, Sara Khadjeh, Christoph Schomburg, Nikola-Michael Prpic

**Affiliations:** 1 Abteilung für Entwicklungsbiologie, Johann-Friedrich-Blumenbach-Institut für Zoologie und Anthropologie, Georg-August-Universität, Göttingen, Germany; 2 Göttingen Center for Molecular Biosciences (GZMB), Ernst-Caspari-Haus, Göttingen, Germany; 3 Present address: Clinic for Cardiology and Pneumology, University Medical Center Göttingen (UMG), Georg-August-University, Göttingen, Germany; 4 Current address: Georg-August-Universität Göttingen, Johann-Friedrich-Blumenbach-Institut für Zoologie und Anthropologie, Abteilung Zelluläre Neurobiologie, 37077 Göttingen, Germany

**Keywords:** Gene duplication, Neofunctionalisation, Spider, Homothorax, Gene expression, Evolution

## Abstract

**Background:**

Gene duplications provide genetic material for the evolution of new morphological and physiological features. One copy can preserve the original gene functions while the second copy may evolve new functions (neofunctionalisation). Gene duplications may thus provide new genes involved in evolutionary novelties.

**Results:**

We have studied the duplicated homeobox gene *homothorax* (*hth*) in the spider species *Parasteatoda tepidariorum* and *Pholcus phalangioides* and have compared these data with previously published data from additional spider species*.* We show that the expression pattern of *hth1* is highly conserved among spiders, consistent with the notion that this gene copy preserves the original *hth* functions. By contrast, *hth2* has a markedly different expression profile especially in the prosomal appendages. The pattern in the pedipalps and legs consists of several segmental rings, suggesting a possible role of *hth2* in limb joint development. Intriguingly, however, the *hth2* pattern is much less conserved between the species than *hth1* and shows a species specific pattern in each species investigated so far.

**Conclusions:**

We hypothesise that the *hth2* gene has gained a new patterning function after gene duplication, but has then undergone a second phase of diversification of its new role in the spider clade. The evolution of *hth2* may thus provide an interesting example for a duplicated gene that has not only contributed to genetic diversity through neofunctionalisation, but beyond that has been able to escape evolutionary conservation after neofunctionalisation thus forming the basis for further genetic diversification.

**Electronic supplementary material:**

The online version of this article (doi:10.1186/s12862-017-1013-0) contains supplementary material, which is available to authorized users.

## Background

Diversification of morphological traits during evolution also requires diversification at the molecular level. Apart from the emergence of entirely new genes [1–3], diversification of gene function can be achieved either through changes at the regulatory level or by alterations in the coding region of the gene. Most genes have more than one function (pleiotropy), thus changes in their coding region and to a lesser extent in their regulatory regions will not only change a single feature of its function, but usually cause multiple (and mostly negative) effects in different organs or tissues (reviewed in [4, 5]). One possibility for overcoming this problem and facilitating evolutionary changes in pleiotropic genes is gene duplication: one copy preserves the original functions of the pleiotropic gene and thus allows the second copy to evolve in an (at least theoretically) almost unrestricted fashion.

Indeed, there is an increasing number of studies that provide evidence for a prominent role of gene duplication in the evolution of physiological or morphological novelty. Especially in plants where duplications of the entire genome are frequent, gene duplications have been implicated in the evolution of morphological novelties [6–8]. But also in animals, gene duplications have been linked to evolutionary novelties, for example in the evolution of spermatogenesis, the immune system, protective embryonic membranes, the physiology of digestion, and the morphology of the exoskeleton in *Drosophila* species [9, 10], sexual dimorphism in stalk-eyed flies [11], the visual system in crustaceans and insects [12], sex determination in bees [13], electric organs and the bulbus arteriosus in fish [14, 15], or the silk producing apparatus and leg morphology in spiders [16, 17].

In the classical model of gene duplication ([18], discussed in [19, 20]), the new copy evolves fast, because it is redundant and therefore is not subject to selection. It thus diverges quickly from the other copy which remains evolutionarily conserved, because of stabilizing selection on the old function. However, if the new copy becomes essential either by subfunctionalisation or neofunctionalisation, it is no longer redundant and from that time point it is also evolutionarily conserved because of its new functionality. The typical outcome of this process is that duplicated genes within a species (paralogs) differ in their expression profile, but the expression pattern of each paralog is evolutionarily conserved between different species.

The genomes of arachnids (including spiders) contain a large number of duplicated genes, and it has already been suggested that these may have fueled the evolution of many arachnid-specific novelties [16, 21, 22]. Previous work has shown that the *homothorax* (*hth*) gene is duplicated in the spider species *Cupiennius salei (C. salei)* and *Acanthoscurria geniculata (A. geniculata)* [23, 24]. The *hth* genes are widely conserved in the Metazoa and homologs have been described from all major arthropod clades [25], onychophorans [26], and from vertebrates, where the gene is called Meis [27]. The *hth* gene of *Drosophila melanogaster (D. melanogaster)* codes for a transcription factor with two highly conserved protein domains. The MEIS domain is a protein-protein binding domain that mediates the binding between Hth and a co-factor named Extradenticle (Exd) [28–30]. In addition to the MEIS domain the Hth protein also contains a modified homeodomain (TALE-HD) that facilitates the binding of Hth to DNA (e.g. [28]). In many cellular contexts Hth first binds to Exd in the cytoplasm, and the protein pair is then translocated to the nucleus where they bind DNA together with Hox proteins [31–35]. Apart from Exd and Hox proteins, Hth is also known to bind other DNA binding proteins (e.g. [36]). Therefore, *hth* is a highly pleiotropic gene and is involved in the formation and function of a large number of organs and tissues, for example leg and antenna specification, eye development, renal tubule growth, muscle fiber identity, and regulation of neuron differentiation (e.g. [31, 37–41]).

The *hth* duplicates in the spiders *C. salei* and *A. geniculata* show a strikingly different expression pattern, especially in the legs. One paralog, termed *hth1*, is expressed in a uniform pattern in the proximal and medial parts of the legs in both species [23, 24, 42]. This expression pattern is similar to *hth* expression in other arthropods and therefore likely is the conserved original expression of the gene before gene duplication [23]. The second paralog, termed *hth2*, is expressed in repeated rings along the proximal-distal axis of the legs in both species. This patterning is not seen in *hth* genes from other animals, and therefore likely represents a novel patterning function after divergence and neofunctionalisation of this second duplicate [23]. Therefore, at first glance, the duplicated *hth* genes in spiders show the typical behaviour of duplicated genes after neofunctionalisation: divergence of expression patterns between the paralogous genes, but strong conservation of the old and the new expression patterns between species. However, the ring pattern of *hth2* is not entirely identical in *C. salei* and *A. geniculata* as noted by Pechmann et al. [43]. We have studied *hth* genes from two additional spider species and show that the expression patterns of *hth2* indeed differ quite substantially between the species. We hypothesise that the *hth2* gene has gained a new patterning function after gene duplication, but has then undergone a second phase of diversification of its new role. The *hth2* gene is apparently a spider specific duplication and not related to other *hth* duplication events of non-spider arachnids. Diversification of *hth2* expression patterns would thus be an interesting example for a duplicated gene that has not only provided genetic diversity through neofunctionalisation, but beyond that has been able to escape evolutionary conservation after neofunctionalisation thus becoming the basis for further genetic diversification.

## Methods

### Animal cultivation and gene cloning

*Parasteatoda tepidariorum (P. tepidariorum)* and *Pholcus phalangioides (Ph. phalangioides)* embryos were obtained from our laboratory stocks in Göttingen, and were treated as described previously [17, 44]. For gene cloning, we used SMARTer RACE cDNA (Clontech, Mountain View, CA, USA) synthesized from a combination of all embryonic stages of the different spider species (for details see [17]). Initial fragments of *Pp-hth1* and *Pt-hth2* were amplified using nested PCR with degenerated primers as described in [23]. Larger gene fragments for *Pp-hth1* were subsequently obtained by RACE PCR using the following primers *Pp-hth-3RACE: GGA CAT CCA TTG TTT CCA TTG TTG G, Pp3RaceHthnew: CAG GGA CTG CGA CGG GGG CCT C. Pp-hth2* was cloned with the following gene specific primers that were designed based on sequence information of a preliminary de novo assembled *Ph. phalangioides* transcriptome (unpublished data):

Pp_hth2_for: CGGTTATCGGGTGGACTTCGG.

Pp_hth2_rev: GGTCCATGATGTTCGGAGGCGAA.

### Phylogenetic analysis

For the phylogenetic analysis of *hth* we searched for larger/complete open reading frames in the transcriptomes of *Ph. phalangioides* (unpublished data) and *P. tepidariorum* [45] using *D. melanogaster* and *C. salei hth* homologs as search queries. With the same query sequences we also searched in the published genome resource of the scorpion *Mesobuthus martensii (M. martensii)* [21], which resulted in three fragments with similarity to *hth*, named MMa38611, MMa00258, and MMa39038 (note that MMa00258 is very short and thus might be an incompletely assembled fragment). For the protein and nucleotide alignments of the *hth* orthologs we used previously published *hth*-related sequences from various metazoan species plus all *hth* sequences identified in the transcriptomes of *P. tepidariorum* and *Ph. phalangioides* and the genome of *M. martensii* (see list of species and accession numbers in Additional file 1).

**Fig. 1:**
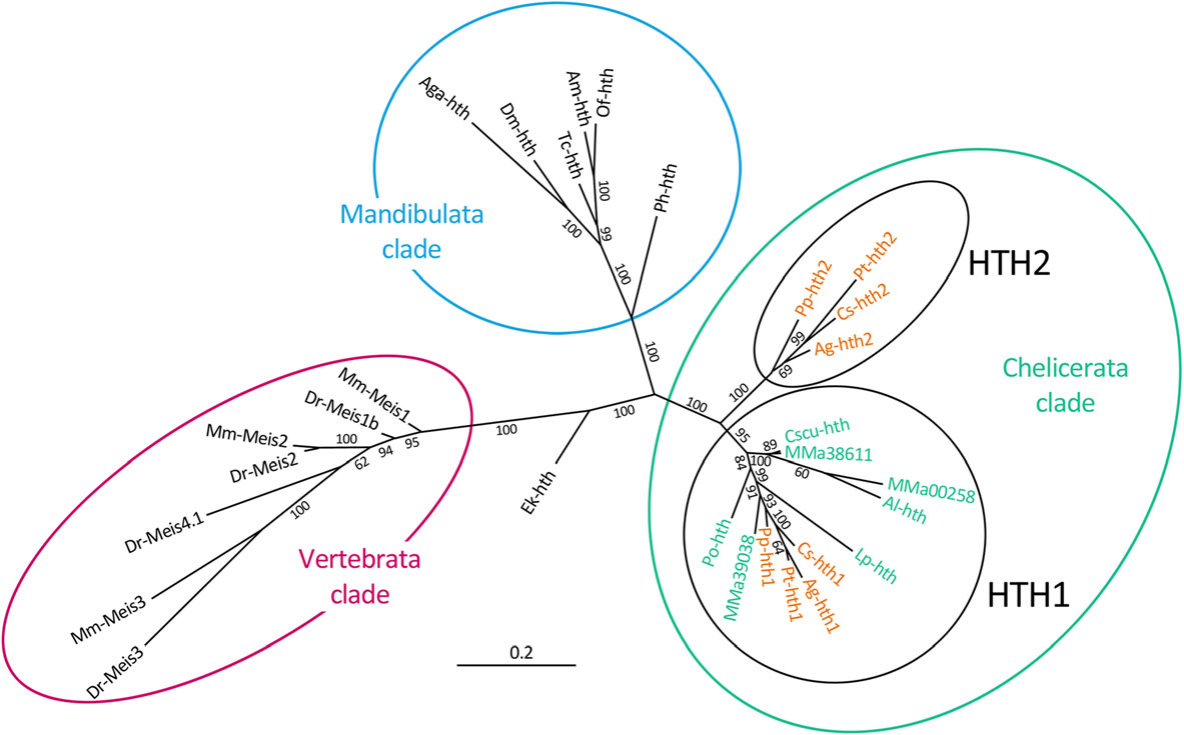
Phylogenetic analysis of *hth* and related protein sequences from diverse Metazoa. Unrooted 50% majority rule consensus tree after Bayesian Markov chain Monte Carlo analysis. Branch lengths in the phylogram give the expected substitutions per site. Numbers at the tree edges are clade credibility values, which are a measure of the probability of each clade in the tree. The monophyletic clades formed by all *Meis* sequences from vertebrates, the *hth* sequences from mandibulate arthropods, and those from chelicerates are indicated by coloured circles (red, blue and green, respectively), Within the Chelicerata clade *hth* sequences from non-spider chelicerates (horse-shoe crab, scorpions, harvestman and mite) are indicated in green, whereas spider sequences are highlighted in *orange*). Homologs of spider *hth1* and *hth2* are indicated by black circles. For species abbreviations and sequence accession numbers please see Additional file 1

Sequences were aligned with Clustal Omega [46] using default settings (the alignments are available in Nexus format in Additional file 2 and Additional file 3). Phylogenetic trees were calculated using the parallel version of MRBAYES (version 3.2.5) [47]. For the protein alignment, after testing mixed amino acid substitution models the Jones model was chosen to generate topological convergence using Metropolis coupling for 179,000 generations after reaching an average standard deviation of split frequencies below 0.01. A total number of 718 trees were written in 2 files (each file contained 359 trees of which 270 were sampled). In the nucleotide sequence analysis (using Metropolis coupling and the 4by4 nucleotide substitution model) an average standard deviation of split frequencies of 0.023742 was reached after 9 million generations. A total of 36,002 trees were written in 2 files (18,001 trees per file of which 13,501 were sampled). The 50% majority rule consensus trees shown in Fig. 1 and Additional file 4 were visualized with Geneious (version 9.1.3) [48].

### Whole mount in situ hybridization and imaging

Fixation and in situ hybridization of embryos were performed according to standard protocols with minor modifications [49]. Digoxygenin-labeled RNA probes were synthesized from RACE PCR fragments for *Pp-hth1*, the entire *Pp-hth2* and *Pt-hth2* fragments, and from the previously published fragment of *Pt-hth1* (GenBank accession number HE608682 [50]). The location of the probes is indicated in Additional file 5. This shows that the probes overlap highly conserved regions and thus in principle could produce unwanted cross-hybridization. However, given the distinct expression patterns detected with the used probes, we think that cross-hybridization was minimal. After in situ hybridization and cell nuclei staining with SYTOX-Green (Invitrogen), whole embryos were imaged with a Leica M205 FA binocular equipped with a QImaging Micropublisher 5.0 RTV camera using combined UV and white light. In addition appendages were dissected and mounted in 80% Glycerol in PBST and images were captured with a Zeiss Axioplan-2 microscope equipped with an Intas digital camera. For colour and brightness correction of all images Adobe Photoshop CS5 Extended or CS6 for Apple Macintosh was used. Staging of embryos was performed on the basis of the published staging schemes for *P. tepidariorum* [51]*, C. salei* [52], and *Ph. phalangioides* [44].

## Results

### Duplicated *hth* genes in *P. tepidariorum* and *Ph. phalangioides*

Previous studies in the entelegyne spider *C. salei* [23] and the bird spider *A. geniculata* [25] have shown that *hth* is duplicated in these species, and that the genes apparently form two separate phylogenetic groups, termed *hth1* and *hth2*, that differ in spatio-temporal expression. To investigate whether the duplication of *hth* is a general feature present in all spider groups, we have studied two additional spider species that belong to two remotely related spider groups, the cellar spider *Ph. phalangioides* and the common house spider *P. tepidariorum*, representing a haplogyne and another entelegyne spider, respectively. At the beginning of the present study transcriptome resources were not available for these species, therefore *hth*-related sequences were amplified from cDNA preparations with degenerated primers and more sequence information was obtained via RACE-PCR (see Methods). As expected from previous studies in spiders we were able to isolate partial sequences of two *hth* paralogs in *P. tepidariorum.* For *Ph. phalangioides* we only identified the *hth1* homolog*.* When transcriptome resources became available, we also searched for *hth*-related sequences in the now available developmental transcriptomes of *P. tepidariorum* [45] and *Ph. phalangioides* (unpublished data). These sequencing resources confirmed the presence of two *hth* homologs in *P. tepidariorum*, and also revealed the presence of a second *hth* homolog in *Ph. phalangioides* that had escaped previous conventional gene cloning attempts. The fact that duplicated *hth* genes are now known from representatives of most major spider lineages including entelegyne spiders, haplogyne spiders and mygalomorph spiders, suggests that this gene is duplicated in all spiders.

### Phylogenetic analysis reveals duplication of *hth* early in spider evolution

We next asked if these duplicates derive from a single duplication event before the radiation of the spider lineages, or if *hth* has been duplicated several times independently in the different spider groups. A previous phylogeny with all then available spider sequences had suggested that the duplicated *hth* genes cluster together according to duplicate and not according to species, suggesting a duplication before the split between mygalomorph and entelegyne spiders [24]. We therefore reconstructed the phylogeny of *hth* and related genes using published *hth* sequences from chelicerates, mandibulates, onychophorans, and the vertebrate Meis genes (see Additional file 1 for a full list of species and sequence accessions). Protein and nucleotide sequences were analysed separately and resulted in phylogenetic trees with very similar topology (Fig. 1, Additional file 4). The vertebrate Meis sequences form a monophyletic group in the trees and are clearly separated from the *hth* sequences from arthropods and the onychophoran. The insect and crustacean sequences also form a monophyletic group that is well separated from the group that contains the chelicerate sequences. Interestingly, the chelicerate sequences are divided into two separate monophyletic groups. The first group (denoted as hth1 in Fig. 1 and Additional file 4) contains all sequences from non-spider chelicerates (including three *hth* fragments (MMa38611, MMa00258, and MMa39038) found in the genome of the scorpion *M. martensii*, see Materials and Methods), the previously described *hth1* copy from *C. salei* and *A. geniculata*, and one *hth* copy each from *P. tepidariorum* and *Ph. phalangioides*, that we therefore also denote as *hth1*. The second group (denoted as hth2 in Fig. 1 and Additional file 4) exclusively contains the *hth2* copy from *C. salei* and *A. geniculata*, and the second copy from *P. tepidariorum* and *Ph. phalangioides* (therefore also denoted as *hth2*).

### Expression of duplicated *hth* genes during embryogenesis

Our phylogenetic analysis suggests that the duplicated *hth* genes in *P. tepidariorum* and *Ph. phalangioides* are orthologs of the *hth1* and *hth2* genes previously described from *C. salei* and *A. geniculata*, and should therefore also show the diverged expression patterns in the legs. To test this assumption, we have studied the expression of the two *hth* genes in *P. tepidariorum* and *Ph. phalangioides* with a focus on the expression during embryonic leg development.

**Fig. 2:**
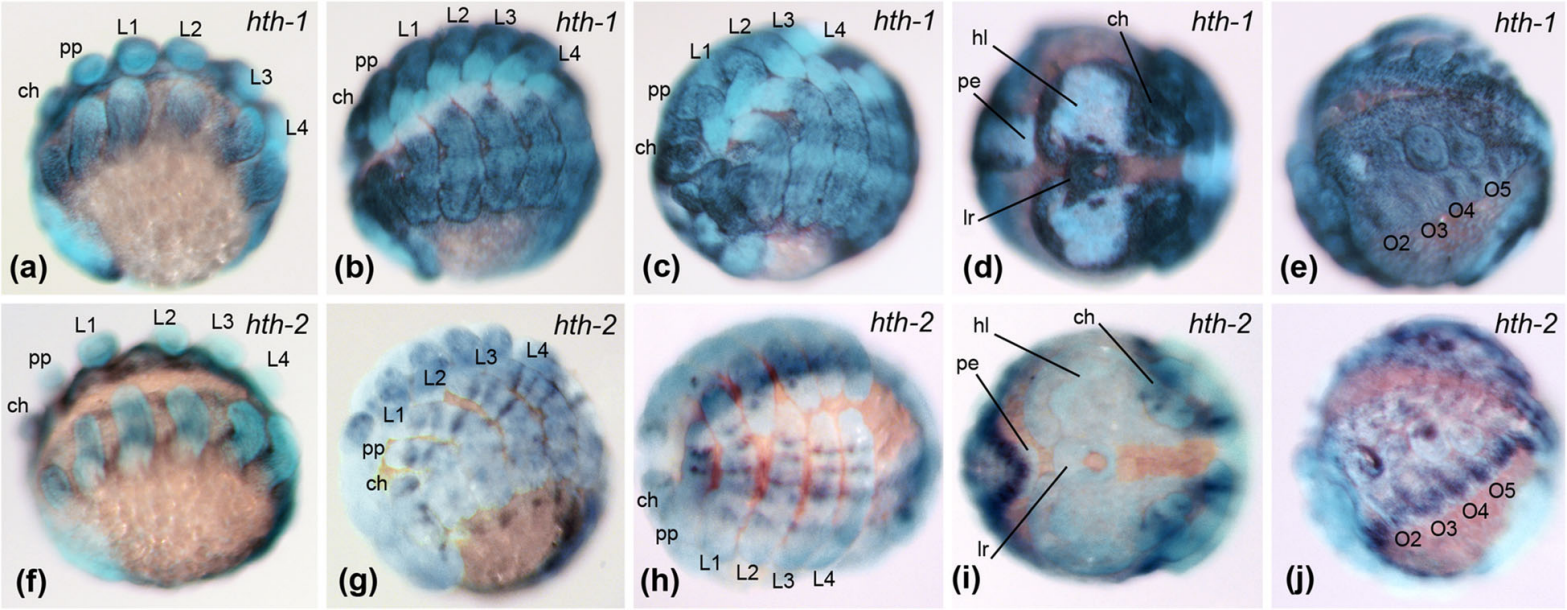
Expression of *hth* paralogs in *Parasteatoda tepidariorum.* Expression of *Pt-hth1* in the prosoma of embryos (**a**) during limb bud elongation, (**b**) inversion, and (**c**) dorsal closure, all in lateral-ventral view. **d** Frontal view of *Pt-hth1* expression in the head of an embryo during inversion. **e** Lateral-ventral view of opisthosomal expression of *Pt-hth1* during inversion. Prosomal expression of *Pt-hth2* (**f**) during limb bud elongation, (**g**) inversion, and (**h**) dorsal closure, all in lateral-ventral view. **i** Frontal view of the head during inversion: no expression of *Pt-hth2* is present in the head. **j** Opisthosomal expression of *Pt-hth2* during inversion, lateral-ventral view. All embryos shown with anterior to the left, except (**d**) and (**i**) which are in frontal aspect. Abbreviations: hl, head lobes; lr, labrum; ch, chelicera; pp., pedipalps; L1-L4, walking legs pairs 1–4; O1-O5, opisthosomal segments 1–5; pe, posterior end of the germband

**Fig. 3:**
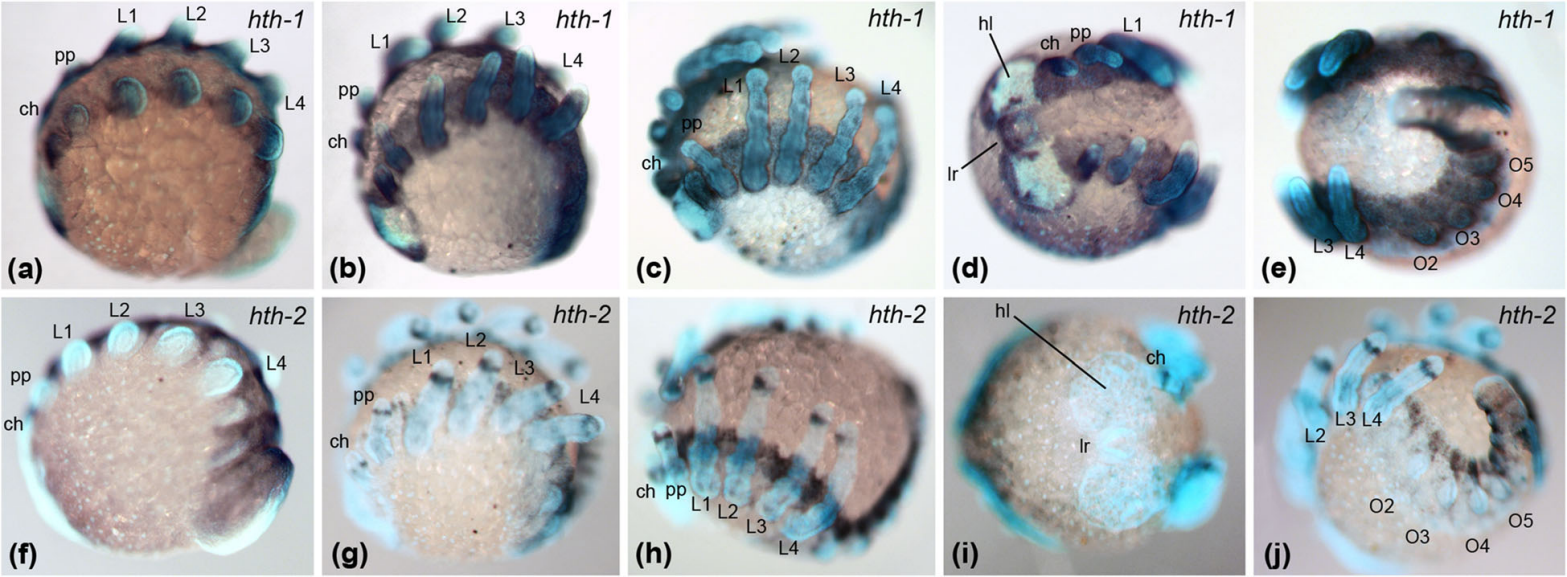
Expression of *hth* paralogs in *Pholcus phalangioides.* Expression of *Pp-hth1* in the prosoma of embryos (**a**) during limb bud elongation, (**b**) inversion, and (**c**) dorsal closure, all in lateral-ventral view. **d** Frontal view of *Pp-hth1* expression in the head of an embryo during inversion. **e** Ventral view of opisthosomal expression of *Pp-hth1* during inversion. Prosomal expression of *Pp-hth2* (**f**) during limb bud elongation, (**g**) inversion, and (**h**) dorsal closure, all in lateral-ventral view. **i** Frontal view of the head during inversion: no expression of *Pp-hth2* is present in the head. **j** Opisthosomal expression of *Pp-hth2* during inversion, lateral-ventral view. All embryos shown with anterior to the left, except (**d**) and (**i**) which are in frontal aspect. Abbreviations: see Fig. 2

**Fig. 4:**
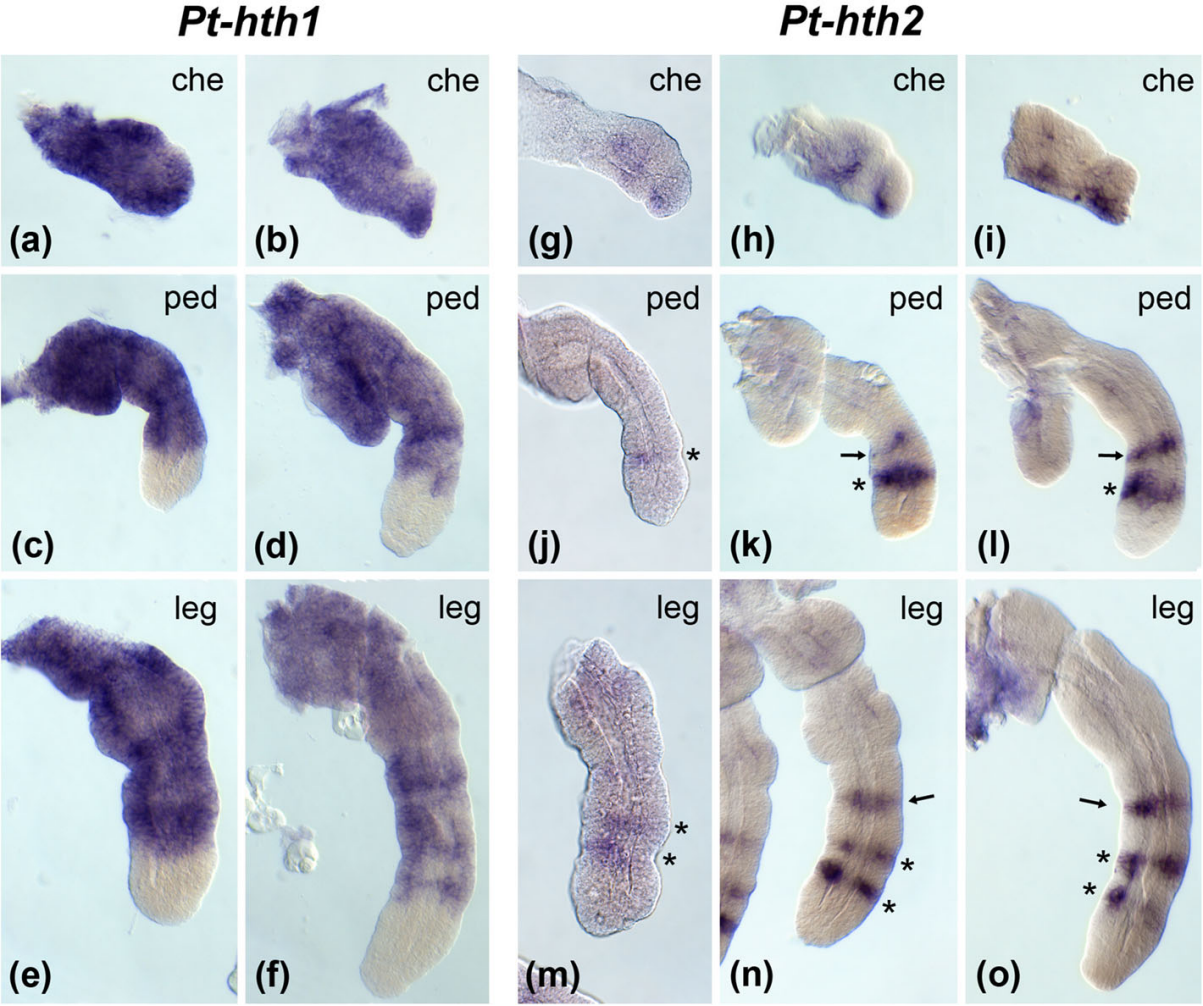
Expression of *hth* paralogs in the prosomal appendages of *Parasteatoda tepidariorum.* **a-f** Expression *of Pt-hth1* is shown in the panels on the left side. **g-o** Expression of *Pt-hth2* is shown in the panels on the right side in the figure. The top row of panels shows dissected chelicerae (che) (**a, g**) at early inversion, (**h**) late inversion, and (**b, i**) dorsal closure, the center row shows dissected pedipalps (ped) (**c, j**) at early inversion, (**k**) late inversion and (**d, l**) dorsal closure, and the bottom row shows dissected walking legs (**e, m**) at early inversion, (**n**) late inversion, and (**f, o**) dorsal closure. The asterisks in (**j-o**) denote distal expression rings (one in the pedipalps, two in the leg). The arrows in (**k, l, n, o**) denote an additional medial ring

**Fig. 5:**
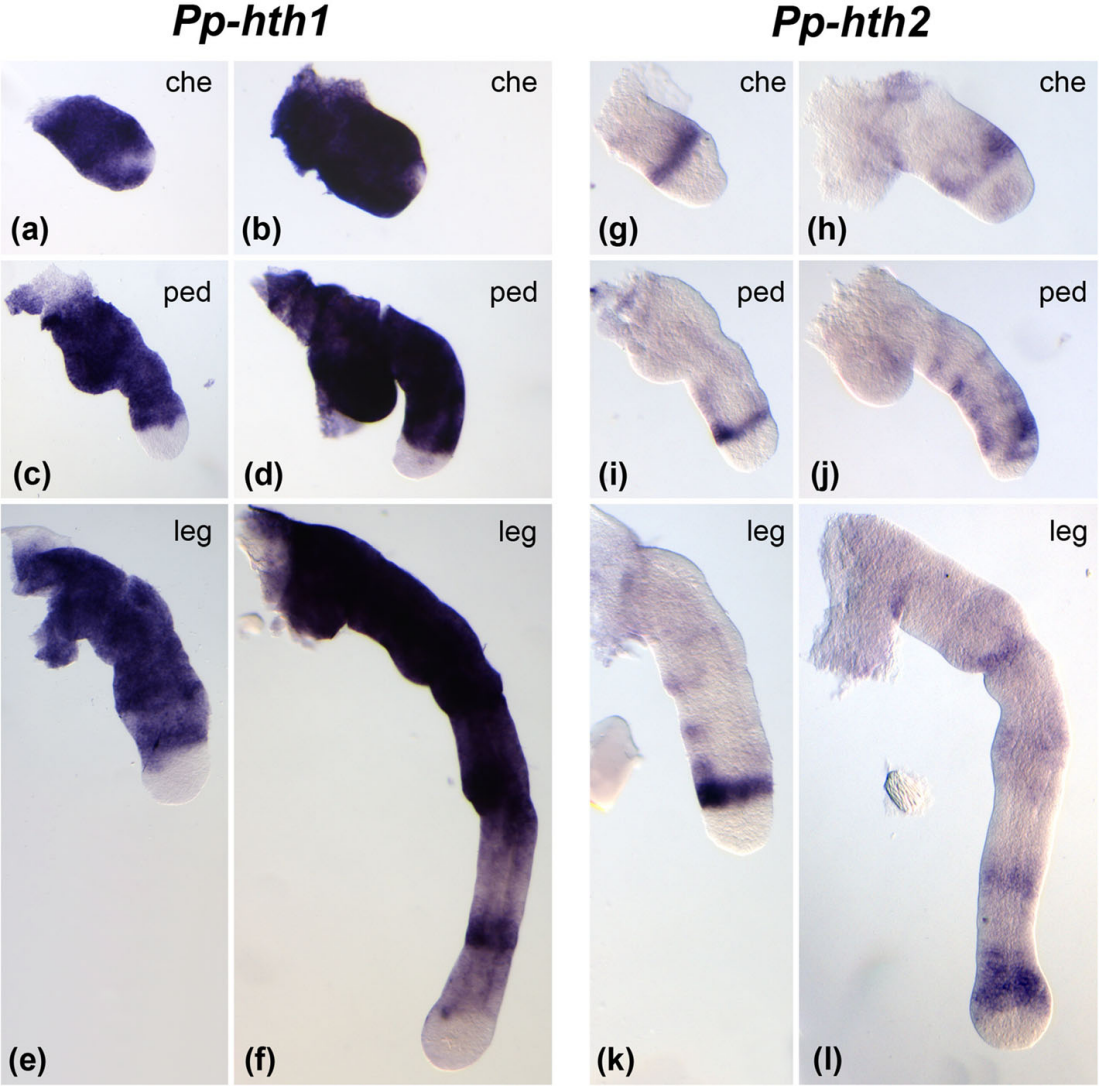
Expression of *hth* paralogs in the prosomal appendages of *Pholcus phalangioides.* **a-f** Expression *of Pp-hth1* is shown in the panels on the left side. **g-l** Expression of *Pp-hth2* is shown in the panels on the right side in the figure. The top row of panels shows dissected chelicerae (che) (**a, g**) at inversion, and (**b, h**) shortly before ventral closure, the center row shows dissected pedipalps (ped) (**c, i**) at inversion, and (**d, j**) shortly before ventral closure, and the bottom row shows dissected walking legs (**e, k**) at inversion, and (**f, l**) shortly before ventral closure

The expression of *hth1* is virtually identical in both species throughout embryonic development, especially in the prosoma. It is strongly expressed throughout the ventral neuroectoderm and in the anterior part of the head. During brain differentiation the expression in the head becomes restricted to the non-neurogenic ectoderm, the labrum, the stomodeum and two stripes next to the stomodeum (Figs. 2d and 3d). When the prosomal limb buds start developing, *hth1* is expressed in the entire prosomal appendages excluding the most distal part (Figs. 2a and 3a). This strong expression in the prosomal appendages remains until late dorsal closure stages (Figs. 2b, c and 3b, c), but then decreases slightly, especially in the distal portion, mainly in the metatarsus (Figs. 4f and 5f). We found minimal differences in the opisthosomal expression patterns between the species. In *P. tepidariorum*, *hth1* is expressed in the entire opisthosoma at uniform levels except for the posterior end and one spot next to the limb bud of the second opisthosomal segment (O2; Fig. 2e). In *Ph. phalangioides* the expression in the opisthosoma is more restricted to presumptive ventral tissue and no expression can be detected in the developing heart tissue (Fig. 3e).

Expression of *hth2* differs from *hth1* in terms of timing and pattern. In both species, the gene is expressed later and in a more restricted fashion than *hth1*. At germ band stages *hth2* is expressed more ventrally than *hth1* in the neuroectoderm and an expression domain in the head tissue or the developing appendages is absent (Figs. 2f, i and 3f, i). First expression of *hth2* in the prosomal appendages is present at early inversion stages, which then differentiates until ventral closure stages (see next chapter), but is always different from *hth1* expression (Figs. 2g, h, and 3g, h). The expression of *hth2* in the opisthosoma differs significantly between *Ph. phalangioides* and *P. tepidariorum*. In *P. tepidariorum hth2* is expressed adjacent to the opisthosomal limb buds, and dorsally in the tissue of the developing heart (Fig. 2j), but in *Ph. phalangioides hth2* is not expressed in heart tissue and expression is restricted to the ventral side of the neuroectoderm and a spot ventral to each opisthosomal limb bud (Fig. 3j).

### Hth expression in the prosomal appendages of different spider species

The largest expression differences between the *hth* duplicates exist in the prosomal appendages (chelicerae, pedipalps and walking legs). To analyse these differences in more detail we studied dissected prosomal appendages of *P. tepidariorum* and *Ph. phalangioides*. Expression of *hth1* is very similar in *P. tepidariorum* and *Ph. phalangioides.* Strong expression of *hth1* can be observed within almost the entire chelicera throughout embryonic development in both species (Figs. 4 a, b and 5 a, b). Throughout most of development *hth1* is also strongly expressed in the proximal and medial domain of the pedipalps and the walking legs, and is excluded from the tip of both appendage types. In embryos undergoing dorsal closure the medial expression becomes weaker and patchy (Figs. 4 c-f and 5 c-f).

Expression of *hth2* is quite different from the pattern of *hth1* and also differs more significantly between the species. In the chelicera of *P. tepidariorum hth2* expression starts as a diffuse domain (Fig. 4g), that later forms two stripes or patches (Fig. 4h, i). In the pedipalps of *P. tepidariorum hth2* expression starts as a weak ring at the distal end of the medial portion of the pedipalp (asterisk Fig. 4j). A second ring appears later in development (arrow in Fig. 4k). In the legs *Pt-hth2* is activated as two weakly expressed rings (asterisks Fig. 4m). An additional ring appears proximal to the initial two rings later in development (Fig. 4n, o (*arrows*)).

In *Ph. phalangioides* the *hth2* expression pattern also comprises ring-shaped domains, but they differ in number and expression dynamics from *hth2* in *P. tepidariorum*. In the chelicera *Pp-hth2* is first expressed as a ring in the medial part, which partially dissolves during development (Fig. 5g,h). In the pedipalp *Pp-hth2* expression starts as one ring in the distal part, and a number of weak segmental rings appear more proximally later in development (Fig. 5i, j). In the walking legs *Pp-hth2* expression also starts as a ring in the distal part (Fig. 5k) and later during development additional weakly expressed rings appear near the future leg joints (Fig. 5l).

**Fig. 6:**
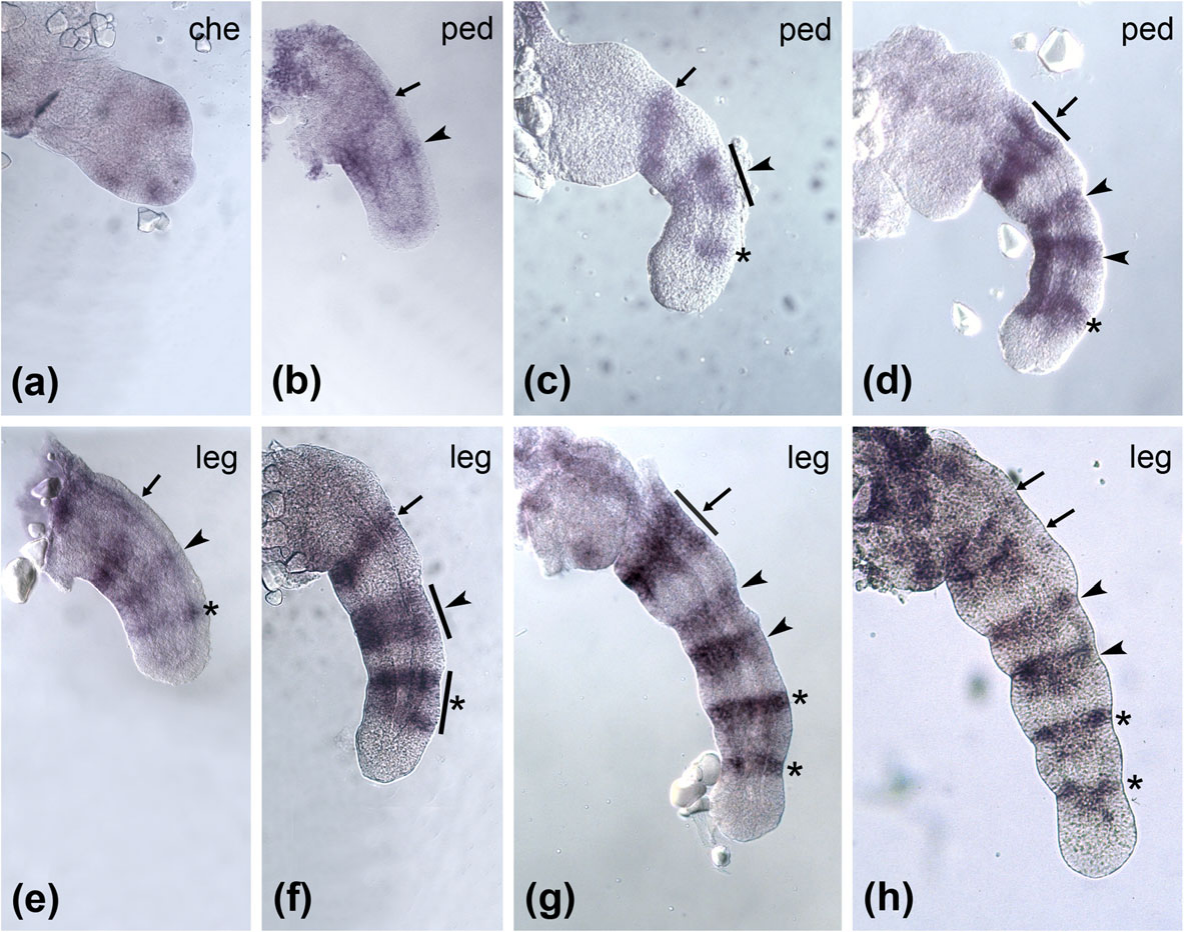
Expression of *hth2* in prosomal appendages of *Cupiennius salei.* **a** Weak expression of *Cs-hth2* in the chelicera during late inversion. Expression of *Cs-hth2* in the pedipalp (**b**) before inversion, (**c**) at early inversion and (**d**) at late inversion. The arrow points to the proximal *Cs-hth2* ring which broadens during inversion (indicated by the bar). The arrowhead points to the medial *Cs-hth2* ring, which broadens during inversion (bar in (**c**)) and splits at late inversion (two arrowheads in (**d**)). The asterisks mark the distal *Cs-hth2* ring. Expression of *Cs-hth2* in the walking legs (**e**) before inversion, (**f**) at early inversion, (**g**) at late inversion, and (**h**) at dorsal closure. The arrows mark the proximal *Cs-hth2* ring and its descendent. The arrowheads mark the splitting medial *Cs-hth2* ring and the asterisks mark the splitting distal *Cs-hth2* ring. Bars always indicate broadening of the ring expression before splitting

We have also re-studied here the expression profile of *hth2* in *C. salei*, because in the original publication, only the expression pattern in the fully formed embryonic leg was shown [23], but the expression profile of this gene is actually more dynamic. *Cs-hth2* has a diffuse expression in the chelicera with a faint medial ring (Fig. 6a). In young pedipalps it is weakly expressed in two rings (arrowhead and arrow, Fig. 6b). Later the proximal of these two rings broadens (arrow, Fig. 6c, d), the medial ring divides (arrowhead(s), Fig. 6c, d), and an additional distal ring appears de novo (asterisk, Fig. 6c, d). In young walking legs *Cs-hth2* is expressed in a proximal, medial and distal ring (arrow, arrowhead and asterisk, respectively, Fig. 6e). All three rings broaden during development (indicated by bars in Fig. 6f, g), and then split into two rings (Fig. 6h). This results in the presence of one ring near each of the developing leg joints in late stages of embryonic development.

## Discussion

### The *hth2* gene has originated by a spider specific *hth* duplication

Duplicated *hth*-related genes exist in vertebrates, scorpions, and spiders. Our phylogenetic analysis indicates that these gene copies do not trace from a single duplication event at the base of the metazoan phylogenetic tree, but have arisen in three independent gene duplication events (Fig. 1; Additional file 4). The ancestral state is the presence of a single *hth*-related gene and this is preserved in the genomes of onychophorans and pancrustaceans. Also a number of chelicerate species have only a single copy of *hth*, which clusters with the *hth1* copy of spiders. This strongly suggests that the spider *hth1* copy preserves the ancestral properties of *hth*, which is also supported by the gene expression pattern (see also below).

Larger numbers of duplicated genes are found in several chelicerate groups including spiders, scorpions and horseshoe crabs [16, 21, 22, 53, 54], but it is still unclear if these trace from a single large-scale gene or genome duplication event in the stem-line of the Chelicerata or if multiple gene duplications have occurred in several chelicerate lineages independently. We have previously shown that the duplication of the *dachshund* gene (*dac*) is an old duplication and has apparently occurred before the diversification of the arachnid orders [17]. Our phylogenetic analysis of *hth* shows that in contrast to *dac*, the *hth* duplicates in scorpions and spiders are placed in separate clades in the phylogenetic tree. Thus, the *hth* duplicates identified in spiders and scorpions are unlikely to trace from the same duplication event and therefore must have been duplicated independently after the split between scorpions and spiders. Genes of the *hth2* type are only found in spiders and therefore represent a relatively young *hth* duplication event that is specific to spiders. The alternative hypothesis that *hth* has been duplicated at the base of the chelicerate or arachnid tree is less parsimonious, because a single *hth* gene is present in other chelicerate groups (including mites and harvestmen) and for these groups additional independent losses must then be assumed.

### Evidence for rapid diversification of *hth2* regulation after gene duplication

Our analysis of the gene expression patterns shows that the regulation of *hth1* is highly conserved, resulting in virtually identical expression patterns in all four spider species. As noted previously [42], this highly conserved expression pattern is comparable with *hth* expression in other arthropods (e.g. [55–58]), onychophorans [26] and vertebrates (e.g. [59]). In addition, not only the regulation, but also the protein coding region of the *hth1* genes is highly conserved: sequence alignments of the spider protein sequences show that the Hth1 amino acid sequences align with high overall identity (Additional file 6). This observation is further corroborated by the phylogenetic sequence analysis that shows that the spider Hth1 sequences group closely together, separated by few expected substitutions per site and thus are closely related phylogenetically (Fig. 1). Taken together, these data strongly suggest that the *hth1* copy retained most of the original gene functions after gene duplication in spiders.

**Fig. 7:**
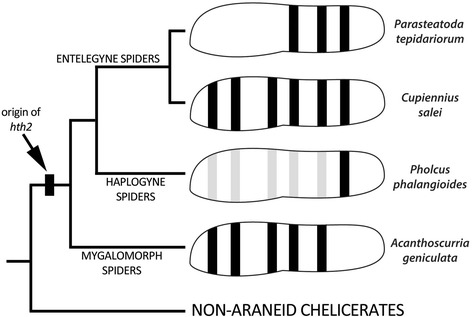
Diversification of *hth2* expression during spider evolution. The drawings show a schematic summary of *hth2* expression in the walking legs in four different spider species (*Acanthoscurria geniculata* after [24]). Black stripes indicate strong expression in the walking legs, light gray bars represent weak expression. The cladogram at the left shows the phylogenetic relationships between the species (phylogeny simplified after [64]). The black box indicates the spider specific duplication event of *hth* that resulted in the origin of *hth2* at the base of the spider phylogenetic tree

By contrast, the *hth2* copy has a markedly different expression profile in most tissues during embryonic development, especially in the prosomal appendages. The pattern in the pedipalps and legs consists of several segmental rings, suggestive of a role of *hth2* in the development of the joints between the limb segments. Surprisingly this role is apparently not required in all segments of all species and each species investigated so far shows a unique pattern of segmental rings (summary in Fig. 7). This pattern does not correlate with the phylogenetic position of the species (e.g. the patterns in *A. geniculata* and *C. salei* are most similar, although both belong to distantly related clades in spider phylogeny). The pattern also does not correlate with the length of the adjacent segments and thus there is no evidence for a role in allometric limb growth. One possibility is that the divergent patters arose by neutral evolutionary processes, e.g. drift, that deleted/reduced different rings in different species. Alternatively, we suggest that expression/lack of expression of *hth2* at the joints might correlate with specific features at certain joints, but the comparative morphology of the limb joints in spiders has not been investigated in such detail to provide direct evidence for this hypothesis. Interestingly, most of the segmental rings of *hth2* are located within the *hth1* expression domain, i.e. *hth1* and the rings of *hth2* (where present) are co-expressed at the limb joints. Thus, apart from a possible new function of *hth2* at the limb joints that would be independent of *hth1*, there is also the possibility that *hth1* and *hth2* act redundantly or cooperatively at selected limb joints to produce a certain morphology. As already mentioned in the introduction, the Hth protein usually binds to the Exd protein to be transferred to the nucleus; however, previous studies have shown that *exd* expression in *C. salei* is restricted to the proximal limb portion and a single distal ring [23]. Thus, most of the *hth2* rings in *C. salei* (and by inference also in the other species) are not co-expressed with *exd* and thus would require other means for their function. We have currently no further data on possible alternative binding factors for *hth2* in spiders, but we note that there is recent evidence that Hth can indeed function independently from Exd [60].

The gain of a novel role of a *hth*-related gene after gene duplication would not be unprecedented. Recent work in bats has shown that a duplicate of Meis, *Meis2*, is implicated in the growth of the wing membrane, a morphological novelty specific to bats [61]. However, the subsequent diversification of the expression pattern after the gain of the new function as seen in the expression patterns of *hth2* in spiders is unusual. Normally genes are expected to conserve their new function after neofunctionalisation and therefore should not show rapid diversification after neofunctionalisation [18]. Our data suggest that genes can escape this pressure of conservation after neofunctionalisation, and thus further diversify. In addition, genuine *hth2* duplicates have so far only been found in opisthothelid spiders that originated in the Triassic period approximately 240 million years ago [62]. This makes the duplication event relatively young in terms of evolutionary age (relative to the age of the entire chelicerata clade which is at least 500 million years old [63]) and suggests that diversification of *hth2* regulation must have occurred rapidly during spider radiation. Interestingly, also the Hth2 proteins (i.e. the coding sequence of the *hth2* genes) from all four spider species studied so far are less similar among themselves than the Hth1 proteins. In sequence alignments the Hth2 amino acid sequences align with less overall similarity (Additional file 6 and Additional file 7), and in the phylogenetic analysis the spider Hth2 sequences are separated by more expected substitutions per site and thus are less closely related phylogentically than are the Hth1 sequences. This suggests that after gene duplication subsequent diversification of *hth2* not only affected its regulatory region, as evidenced by the diverse expression patterns, but to a certain extent also its protein coding region.

## Conclusions

Our study of the duplicated *homothorax* genes in spiders shows that this gene duplication is specific to spiders and that the two copies differ significantly in their expression patterns. In addition, the expression pattern of the *hth2* copy is weakly conserved and shows a different expression pattern in each species studied so far. We conclude from these data that after duplication of *hth* at the base of the spider lineage the *hth1* copy preserved the original functions and therefore shows a pattern similar to the unduplicated *hth* gene in other arthropods. The *hth2* gene on the other hand shows a strongly divergent expression pattern, likely indicating new functionality not shared with the *hth1* copy. We hypothesise that *hth2* after neofunctionalization underwent a second phase of diversification of gene expression that led to the different expression patterns in each species. Thus, *hth2* may serve as an interesting example for a duplicated gene that not only acquired new functionality, but also rapidly diversified this new functionality during evolution.

## Additional files

Additional file 1: Species abbreviations and sequence sources used in the phylogenetic analyses. (DOCX 129 kb)

Additional file 2: Alignment of metazoan *hth*-related protein sequences used in the phylogenetic sequence analysis in Nexus format. (NEX 25 kb)

Additional file 3: Alignment of metazoan *hth*-related nucleotide sequences used in the phylogenetic sequence analysis in Nexus format. (NEX 63 kb)

Additional file 4: Phylogenetic analysis of *hth* and related nucleotide sequences from diverse Metazoa. Unrooted 50% majority rule consensus tree after Bayesian Markov chain Monte Carlo analysis. Branch lengths in the phylogram give the expected substitutions per site. Numbers at the tree edges are clade credibility values, which are a measure of the probability of each clade in the tree. The monophyletic clades formed by all *Meis* sequences from vertebrates, the *hth* sequences from mandibulate arthropods, and those from chelicerates are indicated in the figure. For species abbreviations and sequence accession numbers please see Additional file 1. (JPEG 548 kb)

Additional file 5: Location of the RNA probes indicated on the mRNA sequence of the individual *hth* genes to indicate the overlap with conserved domains. The protein coding sequence (CDS) is shown in bold type, red colour indicates the location of the Meis domain, and blue colour indicates the location of the homeodomain. The location of the probe is mapped onto the sequence by gray background shading. (DOCX 206 kb)

Additional file 6: Alignment of Hth1 proteins from four spider species. Abbreviations: Pp, *Pholcus phalangioides*; Cs, *Cupiennius salei*; Ag, *Acanthoscurria geniculata*; Pt, *Parasteatoda tepidariorum*. Dashes in the alignment denote gaps introduced to improve the alignment. (DOCX 125 kb)

Additional file 7: Alignment of Hth2 proteins from four spider species. Abbreviations: Pp, *Pholcus phalangioides*; Cs, *Cupiennius salei*; Ag, *Acanthoscurria geniculata*; Pt, *Parasteatoda tepidariorum*. Dashes in the alignment denote gaps introduced to improve the alignment. (DOCX 126 kb)
