# Supplementary material for: Rapid diversification of homothorax expression patterns after gene duplication in spiders
Source: BMC Evol Biol. 2017 Jul 14;17:168. doi: 10.1186/s12862-017-1013-0 (PMC5513375; doi:10.1186/s12862-017-1013-0)
Supplement: Supplementary file 1 — Species abbreviations and sequence sources used in the phylogenetic analyses. (DOCX 129 kb) [file 12862_2017_1013_MOESM1_ESM.docx]

**Additional file 1. Species abbreviations and sequence sources used in the phylogenetic analyses.**

| Name | Source (protein) | Source (nucleotide) | Description |
| --- | --- | --- | --- |
| Ag-hth1 | CAS89757 | FM876231 | Acanthoscurria geniculata (Bird spider) hth1, partial |
| Ag-hth2 | CAS89758 | FM876232 | Acanthoscurria geniculata (Bird spider) hth2, partial |
| Aga-hth | XP_308010 | XM_308010 | Anopheles gambiae str. PEST (Mosquito) AGAP002178-PA mRNA |
| Al-hth | AGP03153 | KC513739 | Archegozetes longisetosus (Moss mite) homothorax, partial |
| Am-hth | XP_006564797 | XM_006564734 | Apis mellifera (Honey bee) homothorax-like |
| Cs-hth1 | CAD57739 | AJ518945 | Cupiennius salei (Wandering spider) hth1 |
| Cs-hth2 | CAD57729 | AJ518935 | Cupiennius salei (Wandering spider) hth2 |
| Cscu-hth | AKN63488 | KP129112 | Centruroides sculpturatus (Arizona bark scorpion) homothorax, partial |
| Dm-hth | AAN13474 | NM_057228 | Drosophila melanogaster (Common fruit fly) hth isoform C |
| Dr-Meis1b | NP_571968 | NM_131893 | Danio rerio (Zebrafish) Meis1b |
| Dr-Meis2 | NP_571971 | NM_131896 | Danio rerio (Zebrafish) Meis2 |
| Dr-Meis3 | NP_571853 | NM_131778 | Danio rerio (Zebrafish) Meis3 |
| Dr-Meis4.1 | AAK54829 | AF376049 | Danio rerio (Zebrafish) Meis4.1 |
| Ek-hth | CAX63044 | FN257944 | Euperipatoides kanangrensis (Velvet worm) partial mRNA for homothorax protein (hth gene), isoform A |
| Lp-hth | AKN63487 | KP129111 | Limulus polyphemus (Horseshoe crab) homothorax, partial |
| MMa00258 | Cao et al., 2013 | Cao et al., 2013 | Mesobuthus martensii (Manchurian scorpion) transcript number |
| MMa38611 | Cao et al., 2013 | Cao et al., 2013 | Mesobuthus martensii (Manchurian scorpion) transcript number |
| MMa39038 | Cao et al., 2013 | Cao et al., 2013 | Mesobuthus martensii (Manchurian scorpion) transcript number |
| Mm-Meis1 | NP_034919 | NM_010789 | Mus musculus (House mouse) Meis1 |
| Mm-Meis2 | NP_001153040 | NM_001159568 | Mus musculus (House mouse) Meis2 |
| Mm-Meis3 | AAI17533 | BC117532 | Mus musculus (House mouse) Meis3 |
| Of-hth | AAS93633 | AY584474 | Oncopeltus fasciatus (Milkweed bug) hth |
| Ph-hth | CAO98908 | AM850852 | Parhyale hawaiensis (Beachhopper shrimp) hth |
| Po-hth | AKN63489 | KP129113 | Phalangium opilio (Harvestman) homothorax, partial |
| Pp-hth1 | CUS20076 | LN897690 | Pholcus phalangioides (Cellar spider) hth1 |
| Pp-hth2 | CUS20077 | LN897691 | Pholcus phalangioides (Cellar spider) hth2 |
| Pt-hth1 | CUS20074 | LN897688 | Parasteatoda tepidariorum (Common house spider) hth1 |
| Pt-hth2 | CUS20075 | LN897689 | Parasteatoda tepidariorum (Common house spider) hth1 |
| Tc-hth | NP_001034489 | AJ518941 | Tribolium castaneum (Red flour beetle) hth |
