# Supplementary figures and images for: Rapid diversification of homothorax expression patterns after gene duplication in spiders
Source: BMC Evol Biol. 2017 Jul 14;17:168. doi: 10.1186/s12862-017-1013-0 (PMC5513375; doi:10.1186/s12862-017-1013-0)

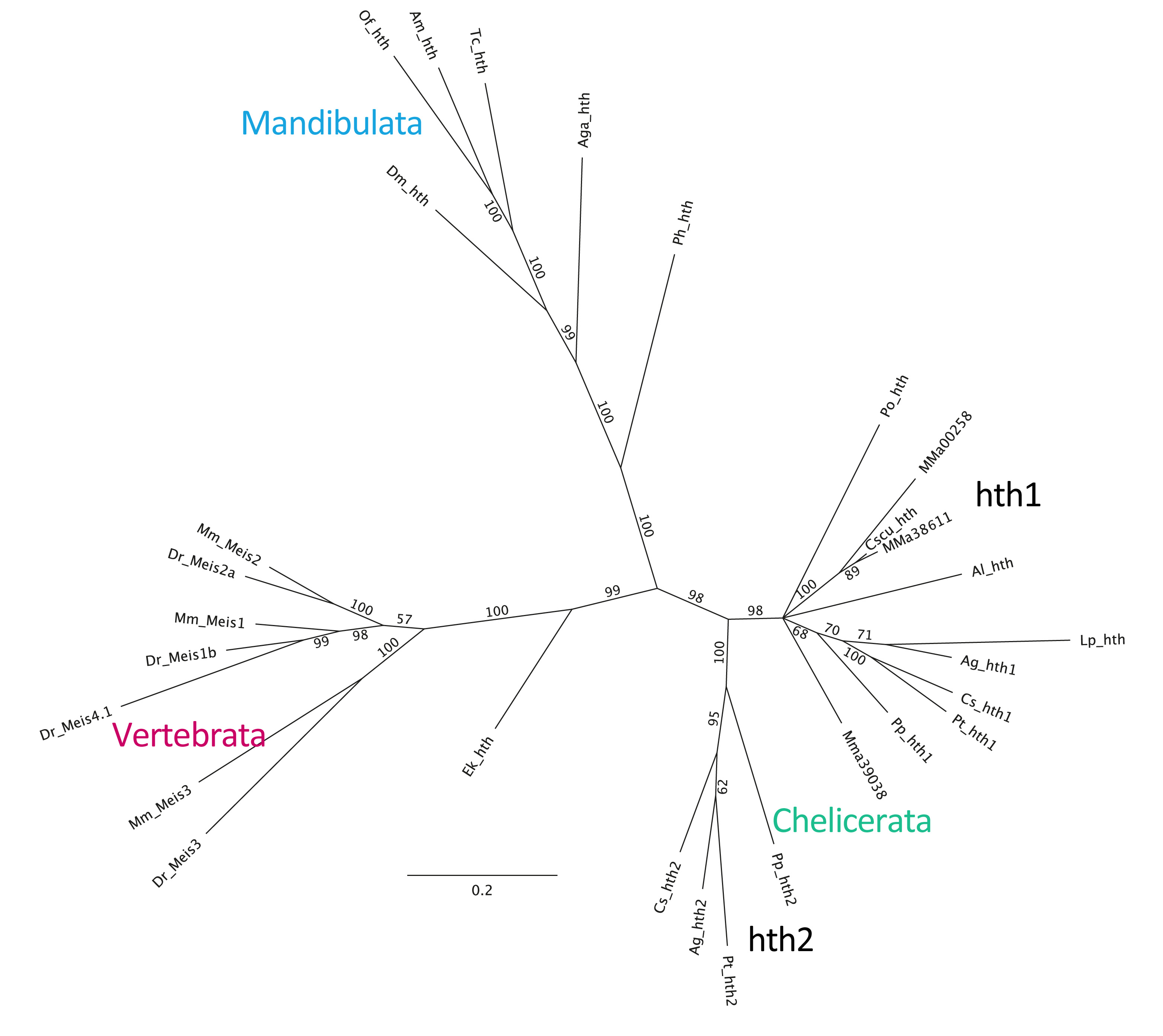

Supplement: Supplementary file 4 — Phylogenetic analysis of hth and related nucleotide sequences from diverse Metazoa. Unrooted 50% majority rule consensus tree after Bayesian Markov chain Monte Carlo analysis. Branch lengths in the phylogram give the expected substitutions per site. Numbers at the tree edges are clade credibility values, which are a measure of the probability of each clade in the tree. The monophyletic clades formed by all Meis sequences from vertebrates, the hth sequences from mandibulate arthropods, and those from chelicerates are indicated in the figure. For species abbreviations and sequence accession numbers please see Additional file 1. (JPEG 548 kb) [file 12862_2017_1013_MOESM4_ESM.jpg]
