# Supplementary material for: Rapid diversification of homothorax expression patterns after gene duplication in spiders
Source: BMC Evol Biol. 2017 Jul 14;17:168. doi: 10.1186/s12862-017-1013-0 (PMC5513375; doi:10.1186/s12862-017-1013-0)
Supplement: Supplementary file 5 — Location of the RNA probes indicated on the mRNA sequence of the individual hth genes to indicate the overlap with conserved domains. The protein coding sequence (CDS) is shown in bold type, red colour indicates the location of the Meis domain, and blue colour indicates the location of the homeodomain. The location of the probe is mapped onto the sequence by gray background shading. (DOCX 206 kb) [file 12862_2017_1013_MOESM5_ESM.docx]

**Additional file 5**

Location of the RNA probes indicated on the mRNA sequence of the individual *hth* genes to indicate the overlap with conserved domains:

**BOLD: CDS**

Red: Meis domain

Blue: homeodomain

Gray background: location of the probe

>Pt_hth1 (Parasteatoda tepidariorum)

UCAGAAAUCUUGGAAGGAUUGCGCCGGACAAUAAGGGACGGUGCGCGGAAUUUUUUCUUUAGCUUGUAGCCGGUGUGUGUUUGUGUAUGUGUUGUCAUUUGCAAGUGAUUUAAGAGGUCAAAGUAUACUUAUAAAAAUAAUAUAAAAAAGCACCGAUUUGAUACCGUUCUUAAAGGAAAACUGGAUUUUUCGUAAGGAAGUUAAUAAACAAAACUUUCCGGAAAGAAAAAAAAAAGUUUUGCAAAGUUUACUUGUUCACCUGUUUCGGCGCAAUCCUUCCAAGAUUUCUGAUUUGUAUAUUAUUUAUUAAAUGCUGUGAUCGAUUUAUUUUGUGAUCCUUGAAGGAUAAAAAUCGUCUGCCAAAAGGAUAUAAUCGUUUGAAAUAUUAAUUUAAAUUAUCUUUUGAGUGGUACGUUUGGUAAGUUAAUCCUUUCGUGCGACAGCUAUCUUUGACAGAUUCUUGUAAUUUGAAUGGCCAUAACAGACCGUUGCUU**AUGAAGAGUGAACCAUACCCAAUUGACCAUUCCCUUGCUAUGCAAUACGAAGAUGGUAUGCCGCACUACGGCGGGAUGGACGGUCCAACGUCCCUAUACGACCCGCACGGGCAUCGGGCAAUGCAACCCCUAUCUCAUGCCCCACAUAUGAAUCACACACCCUCAAUGCACCAGUAUCACAGCAAUCAUGUAUCCAUGUCUAACCAUAUUAUGGGAACUGUACCAGAUGUUCAUAAGAGAGAUAAGGAUGCCAUAUAUGGGCAUCCUCUGUUCCCUCUGCUAGCACUGAUAUUUGAAAAGUGUGAACUGGCCACGUGUACCCCCAGGGAGCCCGGUAUAGCUGGCGGAGAUGUCUGCUCGUCUGAAUCUUUCAAUGAAGACAUAGCUGUCUUCGCCAAACAGAUUAGACAAGAAAAACCAUAUUAUUCUCCCAAUCCCGAGUUAGACAGUCUUAUGGUUCAAGCGAUUCAAGUAUUAAGGUUCCAUUUAUUAGAGUUAGAAAAGGUACACGAAUUGUGCGAUAACUUCUGCCAGCGUUACAUUAGCUGUUUAAAAGGCAAAAUGCCAAUUGAUUUAGUCAUCGAUGAAAGGGACAGCAAACCUGGUGACUUGGGAGACAAUAAUAAUAACAGCAGUAACGGUGGUGGUGGUGCUGGUGGUGGUAACAGUGGGGCAGGAGGUGGAAGGGGUAACCCUGACACCACUGGACACAGCUCAGACAACUCAUCCACUCCAGACCAAUCUUUUAUACCCUACCAGAGACCACCUUCACAAUCUCUAAACUCCUAUAGUACCGGACCUGAUGAUGCCAGAUCACCCGCCGGAUCUACAGGAACUCCAGGCCCUAUUUCACAACAACCUAGUUCACAACUUAGUACUGACAACAACAGUGAAGCAGGUGAUGCGAGUAUCGGGUCUGGUGAUGGAACGGGUGAGGAUGAUGAUGAUGAUAGGAGUAAAAAACGUCAAAAGAAAAGAGGCAUAUUUCCAAAAGUAGCCACAAACAUAAUGAGGGCAUGGCUUUUUCAACAUCUAACGCAUCCCUAUCCAUCCGAAGAUCAAAA**

>Pt_hth2 (Parasteatoda tepidariorum)

CUCCCAACUUCCCAAAAGCGAACAAAUUAUGCAGGAAAGAAAGUUUGUGAUUUUCGCCAACCCUCGCCAAGCAUUCCACGAUUAUUUAUCAUUCCGUUCAAGUUGACGGUGGGUGCAAAGAAAGGGGUGGGUAUAAAAGCGCCCCCUACAGGAAACCGGCUGAGCGAGGGGCGCGAAAUUUUCUCGUGUUGUUUUGAUCGUCAGAUCCAACACGUGGUUGGAAGAGUAACGUUGUCGAC**AUGCAAUAUCCUGAAGAUGGGAUGCCUCAUUACGGCCAUGGGGAUGGAUCGGCCGGCGGUCUAUAUGACCCCCAUAGGCAAAAUCUUAUGAACCACCAUGGUGUUUAUCAUGCCAAUCAUGUGUCUAUUGCUAAUCAUGUCAUGGGAUCUACACCUGAUGUCGGAAAAAGGGAUAAAGAUGCCAUUUUUGGACACCCGCUUUUUCCUCUGUUAGCCUUGAUCUUUGAAAAAUGUGAGCUGGCCACCUGCACCCCUAGGGAACCAGGCAUCGCUGGGGGAGACGUCUGUUCCUCAGAGUCUUUCAAUGAGGAUAUCGCCUGCUUUGCAAAACAGAUUAAGGAAGAAAGACCAUUGUAUGAUGCCAAUCCUGAACUAGAUAGCCUUAUGGUGCAAGCAAUCCAAGUGCUUAGAUUCCAUUUGUUAGAAUUGGAAAAAGUUCAUGAGCUCUGUGAUAACUUUUGUCAGCGUUACAUCAGCUGCCUAAAGGGUAAGAUGCCUAUUGAUUUGGUUAUCGAAGAGAGAGAUACUAAACCUGAGUUAGGGGACACCAAUAACAACAGCAACGGUAGCAGUUUCUGUGGUGGUCCUCCCUGUGUCUCCAGGGGUAUGCUGGAUACUUCCGGCGGACAUAGUACUGAUAGUGGCUCUACACCAGAUCAGGGCCACUAUGAAGACAUGUCUGUGGGAAAUAUGGAAAGGCUAGGGAGACCACCUUCACAAUCCCUGAAUUAUGGUUCCGUAGGCGAUGAUGUUCGGUCCCCCACGGGUUCAACUGGCACUCCAUGUCCUCUGUCACAACAGCCUUCUUCCCAACAAAGUACAGACAAUAACAGUGAAGCUGGUGAUGCGAGUAUUUGUUCAGGAGAAGGCUCAGGUGAUGAGGAUGAUGAUGAAAGGGGUGGUAAGAAAAGGCAGAGGAAGAGGGGUAUUUUUCCUAAAGUAGCGACAAACAUACUGAGGGCAUGGCUAUUUCAGCAUCUUACGCACCCGUAUCCAUCUGAAGAUCAAAAAAAGCAGCUUGCUCAAGACACAGGUCUUACGAUUCUACAGGUAAACAAUUGGUUUAUCAAUGCCCGUAGAAGAAUAGAGUCUUUAAAUUCUGGCUCCAAUUGUACUCAUAUUUUGAGGAUUAAAAUUUUUAAUCCGGCAGGGAAAGCAUAA**CCAUGUUUUUGAGAGGUAAUUGAUCUCUAACACCAAGUAAAAUGCUUAAAAUUUACUCUUCAAACCAUAGUUUAAUAAGUGAAAAUUGUGUCAUUACAGGUUUAUCAAUGCCCGUAGAAGAAUAGUUCAACCUAUGAUUGAUCAGUCAAACAGAGCUGGAGGGACUAUGGGAACACCAGGACCGACAUACAGCCCUGAAUCCUCUGGUAUGGGCUAUAUGAUGGAUGGAGUGUCGCCCAUGCAUAUCAGAUCAUCCAGCCUGCAGAAUUUGCCUUGCCCAGAGAACAUGACCAUUGGCCAUAUGGCAGGAUAUUCUCAGCUCAGGUCACCGGUCCACUCACAAGGAAUGCUUCUUUCUGGACACCCUAUGAUGAUGUCACAUGGUGCGCUACCCCCACCUCCGCAUGGAUCGCCUUAUGAUAGUUCUCCCCCCAGUAUUAUGGACUUGCACAAUAGUUAGUUUAUAAACCAAUAUUCUUGAUAUGGAUAAUUUUGUGUUGUGUAGAAAUUUGUCCAUUUUGUAUACCUCCCCCAAAAAAACCUUAAUCAUGUUCAUUUUUGUAGUGCAAUAAUAAUUUUAUUUCAAGUGUCAUAAAUUUAUUGUUACUGUGGCUCUUCCACUGCAAUAAUGCUCGUCAAUUCUACAGGUUUUAAAAUUAUUAAGUUAAAGAAAGAAUUUUUUUAGCAUAACUUUAGUUCUUAUCCUUUAUUGUUAAUAACUUCGUCUGAAAUUAAACAUAUACGUAUUAACAAUAUAACUUAAAAAACAAUAUUUUAAAAUGCUACAAAUUUUAACCACUUACUUAAAAGAAAUCAAUUAUUUAAAUACAUUUUAGCUGAUAUAUGUUGUUAAUUUUAAUAUAGUUUUUAUCAUUAUAAAUUGUGUAUUUUAUGUUUUUAAAAAAAAUAGUUUGUGUUCAAAUAAUUGUCGGAAUGUUUAGUGAUUUUUGUACAAAUGAAUGGUCAUGUUAAGUUAAAGUGUGUAUCUUCUUAGAAAAAUGUUUUAAAAUGUGAUGAUGAUUGCUGUAAAACGUCUUUGUAUAUAAAUAUAACACUGAAUAAAUUAAUCAGUAUUAAAUUAAAAAAAAA

>Pp_hth1 (Pholcus phalangioides) probe 1

CUAACCAAUCAGCUCUUCCAAACUUUUCCCUUCUUUUUACCUAAAAAUAUCGGAAAAUUUUCAGCACGGGGAGAAGAAAAAGCGCUAACGAAAGAAUUUUAAGAGAAUAAUCAUCUAAUGAAUAAUUUUUAAGACGGAUGAUAAAUUUUAAAUAAAUUAGUAAAAAAAAAAAAUUUAAGUUAAUGAAUUGUAUUUCUUUUUCCACAUUAUUUCCUACCCUUGGCGAAAGGAGAAAGUGGGGAAAGAGAUAGAAGAUAAUCUCCAAGUUGAGUUUCGUCGCCACGGGGAGGCGCUGCCGUCGCCAACUGGCGGUAAAACCACCCACCGGGGAGGGGAAAAAGAGCGCAAAAAGCGCAUUUCCCAUUCAAUUCGCACUUUGCACUUCUUCUCCAUCUCGGCUCCUUCUUAACUUCGCGCGCGUUUAGAUCUUGCAAAAAAAAAUAAAUUAAUUAAUUAAAUAAUUCCUCGCAUUUGAAAAUUAAUUAAUUUAAUCGAUCGACGUAUUUUAUUAUUUAAAAAAAAAAAUUAUUUCAUCCCCUUUUCCCUCCAAGAGGAAAAAAAAAAAAUUAUGAUCGGUAAGAAAAAUUUAUUGGAAAUUUCCUGUGUUUUAUAUCGGACUGGACAUAUAUGAUAUUAUUGUUAUCAUUAUUAUUAUCAUCAUCAUCAUCAUCGAUUUACUGACUGACCUGUGGUGUUGCUGCUGAAAAAAAUUGUUAUUAUUAUUAUAUAUAAUUUUUUUUUCUUCAAAGAAGAGAGAGAAAAAGUGUGAUAACCAGUUUGAAGACCGGUUAGUGGUGUUUCUUGUGUGUGAGUGUGUUUGUGUGUGUUUCUGUGUAUUAUAUUAUAUACACAUAAGUGGCUACUACUACCAUCUUGAAGGAUUUUUUCUUCGACAGAAUAUUUAUUGUACAACAGUUGGUGUUGUUGUUACGGUUGAAGUAAUAACUUCGAUUGAAGAACUAGAUAAGAAUUUGCUGCGAAAAGUGAAAGGAAGCCAUUGAAUCUAUAAAUUGCGUGACAACUUCUCUAAUAUUGGUUUCUUGUGAAAAAAAAGAUAAUAAUAAAAUAAAAGAUUUUAAUAUUGCAGGCAUUAACCUCUUCCGAUCGAUAUAUAUAUAUCUAAAAAAAAGUGAUUCCAGAAGAUUUUUCGUAUUUAUUCACGGGAAAAGAAUCAAUUUUUUGAGAAAUAUUCUAAAAAAAUAAUAAUAGGAUAAUAUUUAAAUUAUAGAGUUGUUUUCAAGUAGUGAAUGUGUGCAGCUUCUUU**AUGAAGAGUGAGCCUUACCCUGUGGAACACCCGUUCGCCACCAUGCAGUACGACGAAGGUAUGUCGCAUUACGGGUCCAUGGACGGACCUGGGUCGCUCUACGAUCCGCAUGGCCACAGUAGGGCCAUGCAAUCGCUGGGACACGCUCCACAUAUGAAUCACACACCAUCUAUGCAUCAAUACCACGGUAAUCAUGUGUCCGGUGUCAUGUCCAAUCAUAUUAUGGGUUCCGUACCCGAUGUUCACAACAAGCGCGACAAAGACGCUAUAUACGGACAUCCAUUGUUUCCAUUGUUGGCUCUAAUUUUUGAGAAAUGCGAAUUAGCUACGUGUACUCCACGAGAGCCUGGAAUUGCUGGUGGAGAUGUUUGCUCUUCGGAAUCUUUCAAUGAAGACAUUACAGUAUUUGCCAAACAGAUAAGGCAAGAAAAACCAUAUUAUGCCCCAAAUCCCGAAUUAGACAGCCUUAUGGUACAAGCUAUUCAGGUCUUGAGAUUCCAUCUGUUGGAACUGGAAAAGGUGCACGAGUUAUGCGACAACUUCUGUCAACGAUAUAUCAGUUGCCUGAAAGGUAAGAUGCCCAUCGAUUUGGUCAUCGACGAAAGAGAUUCCAAACCGGGUGAUCUCGGUGACAACAACAACAGCAGCAAUGGCGGCAGUGGCAAUGGUGGUGGCGCCGGUGGUGGAAGCGGUGGUGCUGGUGGCAAUGGUGGCAGAGGCAAUCCCGAUACGACUGGUCACAGUUCGGACAAUUCGUCGACGCCUGACCAGAGGCCCCCGUCGCAGUCCCUGAAUUCGUACAGCACAGGAGGAGAGGAUGCAAGGUCGCCCGCUGAUUCGACAGGGACACCGGGACCUAUAUCCCAACAACCGAGUUCGCAGAUGAGCACCGACAACAACAGUGAAGCCGAUGUCUCGACUUACGGCGAUGCUAGUAUUGGAUCCGGAGAUGGAACCGGCGAAGACGACGACGACGACAGAAGUAAGAAACGCCAGAAGAAGAGAGGAAUCUUCCCGAAGGUCGCCACGAAUAUCAUGAGAGCCUGGCUCUUCCAGCAUCUUACGCAUCCAUACCCUUCUGAAGAUCAGAAGAAGCAACUUGCUCAAGAUACUGGUCUGACGAUUCUGCAAGUGAAUAAUUGGUUUAUCAAUGCAAGACGGCGAAUAGUUCAGCCUAUGAUUGACCAAUCCAACAGAGCUGGUGGAGCUACAGCUGCGUACGGACCCGAUGGCGCAGGUAUGGGAUACAUGAUGGAUGGAAGCCAGCAGAUGCACAUCCGCCCACCAGGUAUGCAGAAUCUUUCAUGCAGUGACGGAGCCAUGGGCAUGGGCCACAUGGGAGGAAUGGGCGGAUAUUCUCAGAUGUCUCAGCUGCGUUCACCCGUUCACUCGCAAGCAAUGCUUCUUCCGGGACAUCCCCACGCUAUGAUGAUGGCGCACGGCCCCAUGGGACAUCCCGGAUUGCCACCGCAAGGAUCUCCGUACGACGGUUCCGGUGGGCACAUCAUGGAUAUCCACGCUAGUUAA**ACAAUUUCAACUUACAAGAAACAAACAAUUAUACGACAAUAACAACAAAAAACCCUUUUCUCCUCAAAAAUGGGGCUAUUGCUAAAAAAAAAAAAAUGAAAAUAAAUCGGCAAGUAGCCAUUUUUUUCCUCGGAGCUUAUAUCACACAAACAAGUCCCUUUUUAAAAAAUGAAAAAUACAAAAAUUUAGGGAAAAAAAAUUUUUUAGAGGAAAAAAAUUCAUUGUUUCCAACAGGAAAAAAAAAUAAUUAAACUAUAAUUUUUAAGAUUUUUCCCACCUCAUCUACCGCAAAAAUUUUUAACCCUCUUCCCAAAAAAUUCCCAUAAAAAUUAUGUCUCUGGAAUUUCAUGGGAAAUGCAAAACUAUAGAACAAAAAAAUACUUACAUGUUGCCGAAAAUAAUUAUUAUAACUAUAAAAUUAAACAUGAAUCUCCCAAAAAAAAUUUUUUUGUGCUUGAUUGAAACAUUCGAAUUAUUAUUUUGUGUUUCUAAAUGGCCAUAAUUAUAUAUAACCCUUCUCUUUAUAGAUGUGCCGUUGUAAUAAUAAUGAUAAAUAAUGUGUAAUGAUAAUUAUUAUCUCUACUCUACAUGUCGAUGCCACGUGUGUGUGUAUAAUGGAUAAUGUAUUUUAUCUCUUAUCUCUAUUUGAUCAUGAUAAAAAUAAUUGAUUGUCCAGUCCAAUCAGAUGAAAAACGAACUCUUGAAUGAUGAUGAUGCUCCGUUAAUUAUUAUUAUUAAUUAUUAUUAUAGAUGACAAUUGUUUGCUGUUGAGUUUUGAGAAUUGAGACUGUUGGCCAGUUCUUUAUAUGUAAUAUAAUACACAUUAUAUUUCAUUCGGUGUAAUUUUACUUCCCCUUUACCCCGAGAAUUCAUUGUGGUCAUGUCGGUAUGGACAUUAUUUGAAAUAAUGUUGAAAUUUAAAAAGAAUAGACAAUACUGCAUAUAUAAUAUACAAUACUGUAUCUUCUCGCUCGCUUUUUCUGUUCUUAUUUUGGAGCUGCACAUUUUUUCAAUGUUGUUGUAAUAAUUUAUAAAAAUAAUCAUGUGUUACGGCGUGUUAUUUUUUAUUGGUUUUUUGCAAAUUUACCAAGGAACAAAAAAAAAAAAACGGUCGUUUUUACAUUUUUUCUCGAUUUUUACAAUAGUGAUUAGAUAUUGAUGAUUAUUGCCACUUACUGCUGCUGCAAUAUAAUUUUAAAUAAUUAAUGAUUUUAAUUAAAAUAAUGCUCAUUAUUAUUUUAUCUAUGAUGGUGCUGUGAUUGAUUCCGUUUAUUUCUUAUGGAGACAUUAUUAGCGAUGGAUAUAAUGAUUUUUUUUUUUACCCCCGUUAUUGUUUUAAUCAUGGCACUUAUUAAUUAUUAUAUUAAUGUUAUACAGUAUAUUUUUUUUUCAGUUUUGAUGGGAAUAAUUAGAAAUAUUUGAAUUGGAAAUUAUUAUUAUUAUUUCUUCUGUCCGUUUGAUUAUAUAUAUAACGGUGGUAAUCUGUAUAGAACUUUUCUCAUAUAGGUGACUCUGCAUUUGUAUGUGACGUGCUUAUGAUUGUAUUGUUGUAACCUGUUAAAUUAAAAAAAAAUAUUCAAAAAUAAAAGUGAAAUGUUAAAAAAAAA

>Pp_hth1 (Pholcus phalangioides) probe 2

CUAACCAAUCAGCUCUUCCAAACUUUUCCCUUCUUUUUACCUAAAAAUAUCGGAAAAUUUUCAGCACGGGGAGAAGAAAAAGCGCUAACGAAAGAAUUUUAAGAGAAUAAUCAUCUAAUGAAUAAUUUUUAAGACGGAUGAUAAAUUUUAAAUAAAUUAGUAAAAAAAAAAAAUUUAAGUUAAUGAAUUGUAUUUCUUUUUCCACAUUAUUUCCUACCCUUGGCGAAAGGAGAAAGUGGGGAAAGAGAUAGAAGAUAAUCUCCAAGUUGAGUUUCGUCGCCACGGGGAGGCGCUGCCGUCGCCAACUGGCGGUAAAACCACCCACCGGGGAGGGGAAAAAGAGCGCAAAAAGCGCAUUUCCCAUUCAAUUCGCACUUUGCACUUCUUCUCCAUCUCGGCUCCUUCUUAACUUCGCGCGCGUUUAGAUCUUGCAAAAAAAAAUAAAUUAAUUAAUUAAAUAAUUCCUCGCAUUUGAAAAUUAAUUAAUUUAAUCGAUCGACGUAUUUUAUUAUUUAAAAAAAAAAAUUAUUUCAUCCCCUUUUCCCUCCAAGAGGAAAAAAAAAAAAUUAUGAUCGGUAAGAAAAAUUUAUUGGAAAUUUCCUGUGUUUUAUAUCGGACUGGACAUAUAUGAUAUUAUUGUUAUCAUUAUUAUUAUCAUCAUCAUCAUCAUCGAUUUACUGACUGACCUGUGGUGUUGCUGCUGAAAAAAAUUGUUAUUAUUAUUAUAUAUAAUUUUUUUUUCUUCAAAGAAGAGAGAGAAAAAGUGUGAUAACCAGUUUGAAGACCGGUUAGUGGUGUUUCUUGUGUGUGAGUGUGUUUGUGUGUGUUUCUGUGUAUUAUAUUAUAUACACAUAAGUGGCUACUACUACCAUCUUGAAGGAUUUUUUCUUCGACAGAAUAUUUAUUGUACAACAGUUGGUGUUGUUGUUACGGUUGAAGUAAUAACUUCGAUUGAAGAACUAGAUAAGAAUUUGCUGCGAAAAGUGAAAGGAAGCCAUUGAAUCUAUAAAUUGCGUGACAACUUCUCUAAUAUUGGUUUCUUGUGAAAAAAAAGAUAAUAAUAAAAUAAAAGAUUUUAAUAUUGCAGGCAUUAACCUCUUCCGAUCGAUAUAUAUAUAUCUAAAAAAAAGUGAUUCCAGAAGAUUUUUCGUAUUUAUUCACGGGAAAAGAAUCAAUUUUUUGAGAAAUAUUCUAAAAAAAUAAUAAUAGGAUAAUAUUUAAAUUAUAGAGUUGUUUUCAAGUAGUGAAUGUGUGCAGCUUCUUU**AUGAAGAGUGAGCCUUACCCUGUGGAACACCCGUUCGCCACCAUGCAGUACGACGAAGGUAUGUCGCAUUACGGGUCCAUGGACGGACCUGGGUCGCUCUACGAUCCGCAUGGCCACAGUAGGGCCAUGCAAUCGCUGGGACACGCUCCACAUAUGAAUCACACACCAUCUAUGCAUCAAUACCACGGUAAUCAUGUGUCCGGUGUCAUGUCCAAUCAUAUUAUGGGUUCCGUACCCGAUGUUCACAACAAGCGCGACAAAGACGCUAUAUACGGACAUCCAUUGUUUCCAUUGUUGGCUCUAAUUUUUGAGAAAUGCGAAUUAGCUACGUGUACUCCACGAGAGCCUGGAAUUGCUGGUGGAGAUGUUUGCUCUUCGGAAUCUUUCAAUGAAGACAUUACAGUAUUUGCCAAACAGAUAAGGCAAGAAAAACCAUAUUAUGCCCCAAAUCCCGAAUUAGACAGCCUUAUGGUACAAGCUAUUCAGGUCUUGAGAUUCCAUCUGUUGGAACUGGAAAAGGUGCACGAGUUAUGCGACAACUUCUGUCAACGAUAUAUCAGUUGCCUGAAAGGUAAGAUGCCCAUCGAUUUGGUCAUCGACGAAAGAGAUUCCAAACCGGGUGAUCUCGGUGACAACAACAACAGCAGCAAUGGCGGCAGUGGCAAUGGUGGUGGCGCCGGUGGUGGAAGCGGUGGUGCUGGUGGCAAUGGUGGCAGAGGCAAUCCCGAUACGACUGGUCACAGUUCGGACAAUUCGUCGACGCCUGACCAGAGGCCCCCGUCGCAGUCCCUGAAUUCGUACAGCACAGGAGGAGAGGAUGCAAGGUCGCCCGCUGAUUCGACAGGGACACCGGGACCUAUAUCCCAACAACCGAGUUCGCAGAUGAGCACCGACAACAACAGUGAAGCCGAUGUCUCGACUUACGGCGAUGCUAGUAUUGGAUCCGGAGAUGGAACCGGCGAAGACGACGACGACGACAGAAGUAAGAAACGCCAGAAGAAGAGAGGAAUCUUCCCGAAGGUCGCCACGAAUAUCAUGAGAGCCUGGCUCUUCCAGCAUCUUACGCAUCCAUACCCUUCUGAAGAUCAGAAGAAGCAACUUGCUCAAGAUACUGGUCUGACGAUUCUGCAAGUGAAUAAUUGGUUUAUCAAUGCAAGACGGCGAAUAGUUCAGCCUAUGAUUGACCAAUCCAACAGAGCUGGUGGAGCUACAGCUGCGUACGGACCCGAUGGCGCAGGUAUGGGAUACAUGAUGGAUGGAAGCCAGCAGAUGCACAUCCGCCCACCAGGUAUGCAGAAUCUUUCAUGCAGUGACGGAGCCAUGGGCAUGGGCCACAUGGGAGGAAUGGGCGGAUAUUCUCAGAUGUCUCAGCUGCGUUCACCCGUUCACUCGCAAGCAAUGCUUCUUCCGGGACAUCCCCACGCUAUGAUGAUGGCGCACGGCCCCAUGGGACAUCCCGGAUUGCCACCGCAAGGAUCUCCGUACGACGGUUCCGGUGGGCACAUCAUGGAUAUCCACGCUAGUUAA**ACAAUUUCAACUUACAAGAAACAAACAAUUAUACGACAAUAACAACAAAAAACCCUUUUCUCCUCAAAAAUGGGGCUAUUGCUAAAAAAAAAAAAAUGAAAAUAAAUCGGCAAGUAGCCAUUUUUUUCCUCGGAGCUUAUAUCACACAAACAAGUCCCUUUUUAAAAAAUGAAAAAUACAAAAAUUUAGGGAAAAAAAAUUUUUUAGAGGAAAAAAAUUCAUUGUUUCCAACAGGAAAAAAAAAUAAUUAAACUAUAAUUUUUAAGAUUUUUCCCACCUCAUCUACCGCAAAAAUUUUUAACCCUCUUCCCAAAAAAUUCCCAUAAAAAUUAUGUCUCUGGAAUUUCAUGGGAAAUGCAAAACUAUAGAACAAAAAAAUACUUACAUGUUGCCGAAAAUAAUUAUUAUAACUAUAAAAUUAAACAUGAAUCUCCCAAAAAAAAUUUUUUUGUGCUUGAUUGAAACAUUCGAAUUAUUAUUUUGUGUUUCUAAAUGGCCAUAAUUAUAUAUAACCCUUCUCUUUAUAGAUGUGCCGUUGUAAUAAUAAUGAUAAAUAAUGUGUAAUGAUAAUUAUUAUCUCUACUCUACAUGUCGAUGCCACGUGUGUGUGUAUAAUGGAUAAUGUAUUUUAUCUCUUAUCUCUAUUUGAUCAUGAUAAAAAUAAUUGAUUGUCCAGUCCAAUCAGAUGAAAAACGAACUCUUGAAUGAUGAUGAUGCUCCGUUAAUUAUUAUUAUUAAUUAUUAUUAUAGAUGACAAUUGUUUGCUGUUGAGUUUUGAGAAUUGAGACUGUUGGCCAGUUCUUUAUAUGUAAUAUAAUACACAUUAUAUUUCAUUCGGUGUAAUUUUACUUCCCCUUUACCCCGAGAAUUCAUUGUGGUCAUGUCGGUAUGGACAUUAUUUGAAAUAAUGUUGAAAUUUAAAAAGAAUAGACAAUACUGCAUAUAUAAUAUACAAUACUGUAUCUUCUCGCUCGCUUUUUCUGUUCUUAUUUUGGAGCUGCACAUUUUUUCAAUGUUGUUGUAAUAAUUUAUAAAAAUAAUCAUGUGUUACGGCGUGUUAUUUUUUAUUGGUUUUUUGCAAAUUUACCAAGGAACAAAAAAAAAAAAACGGUCGUUUUUACAUUUUUUCUCGAUUUUUACAAUAGUGAUUAGAUAUUGAUGAUUAUUGCCACUUACUGCUGCUGCAAUAUAAUUUUAAAUAAUUAAUGAUUUUAAUUAAAAUAAUGCUCAUUAUUAUUUUAUCUAUGAUGGUGCUGUGAUUGAUUCCGUUUAUUUCUUAUGGAGACAUUAUUAGCGAUGGAUAUAAUGAUUUUUUUUUUUACCCCCGUUAUUGUUUUAAUCAUGGCACUUAUUAAUUAUUAUAUUAAUGUUAUACAGUAUAUUUUUUUUUCAGUUUUGAUGGGAAUAAUUAGAAAUAUUUGAAUUGGAAAUUAUUAUUAUUAUUUCUUCUGUCCGUUUGAUUAUAUAUAUAACGGUGGUAAUCUGUAUAGAACUUUUCUCAUAUAGGUGACUCUGCAUUUGUAUGUGACGUGCUUAUGAUUGUAUUGUUGUAACCUGUUAAAUUAAAAAAAAAUAUUCAAAAAUAAAAGUGAAAUGUUAAAAAAAAA

>Pp_hth2 (Pholcus phalangioides)

UAAAAAGUUAACAGAAACCAAACUCCUGUUAUUACACGCACUACACGGAAAAACUUGGUCGGGUGAAGUCGCUUUCACGCGCGAGCGCCAUCUGUGAGAAUUCCGCGGCGGCACCUUCCACAAGUUUAUCCGCCGGUUAUCGGGUGGACUUCGGUGGUGACCAUUUCUCUCGUGUUUAUUUGUGUCUUUUUUUUCAAUCGUUGAAUAAAUUGGUUUCGGGCUUUCCUUUGGAAUAAUAAAAUAACGACGUACGAUA**AUGCAGUAUAACGAAGACGGCAUCCCUCACCCUUACGGAGUGGACGGUGGCGGGCCGCCGUCCCUUUACGACCCGCACCGGCCGAUGCCGAACCUGUCCCAUCACAUGAACCACGGACCGUCCAAUAAUCUCCAUCAGUAUGGCAACAGUCAUGUCAACAUCGCCAACCACGUCAUGGGGUCCAUGCCGGACGUUCACAAACGGGACAAAGACGCUAUAUACGGGCAUCCAUUGUUUCCAUUGUUGGCGCUAAUCUUCGAAAAAUGUGAACUGGCCACUUGUACGCCGAGGGAACCAGGCAUACCCGGCAACGAUGUCUGUUCAUCGGAGUCAUUCAACGAAGACAUCGCAGUUUUUGCCAAACAGAUUAGGCAGGAGAGACCUUACUACAGCCCCGACGAGGAACUCGACAGCAUCAUGGUACAAGCAAUCCAAGUCUUGAGAUUCCAUCUAUUGGAAUUGGAGAAAGUCCACGAGCUGUGCGACAAUUUCUGCCAGAGAUACAUCAGCUGCCUGAAGGGCAAGAUGCCCAUAGAUUUGGUCAUCGAGGAGAGGGACACCAAACCCGAACUCGGCGACACCAACAACAACAGCAACGGCAGCAGCUACUGCGGGGGCCCGCCGUGUGUCCCCAGGGGUAUGAUGGACACUUCGGGACACAGCACGGACAGCGCGUCCACUCCUGAUCAGAGACCACCAUCGCAGAGUUUGAAUUACGGUCCGGUCAGCGAUGACGUCCGGUCGCCGGCGUCCGCGGGCACACCCUGCCCAUUGUCCCAACAGCCCGCGUCCCAGCAGAGCACGGACAACAACAGCGAAGUCGGUGAGUGGGACGCCAGCAUCGGAUCUGGAGAAGGGACCGGCGACGAAGACGACGACGACAGAGCCAAGAAGAACCAGAAGAAACGUGGGAUAUUCCCCAAGGUCGCUACUAACAUCAUGAGGGCUUGGCUCUUCCAGCAUCUCACGCAUCCAUAUCCAUCCGAGGACCAGAAGAAGCAGCUCGCUCAGGAUACGGGUCUCACGAUCCUUCAAGUCAACAAUUGGUUCAUCAAUGCCAGGCGUAGAAUUGUACAGCCGAUGAUUGAUCAGUCGAACAGAGCAGGUGGCAGUAUCGGUCCACCAGGAGCGUCUUACAGCCCGGAGUCUUCUAUGGGAUACCUGAUGGAUGGUGUGCCGCAGAUGCAUAUACGACCAGGUCUCCAGGGAUUGCCGGAUUCGUCGAUGGGUCACAUGGGCUACUCCCAGCUCCGUUCUCCUGUCCAUUCCCAGGCCAUGCUGAUCCCGGGACACCACGCCAUGAUGAUGUCUCACCCUGGGUUGCCGCCUCCACCUCCUCACGGAUCGCCUUAUGACAGUUCGCCUCCGAACAUCAUGGACCUUCACUCGAGCUGA**GACUGUUCACUUCAAAAAUAUUAUAAAUUCACAAUAUAUAUAUAAUAUAUGUAUCUCGAUGUGUGAAAGAGAUCGUUAUGCGUGACUUAAUAUAACAUAGAUUUGUGGCGCCAUCUAUAUAUAUGCUACCAGUUUUUUUUUUGUCUUGCUUUCUUUCCCAUUGCACUCAACUCACUCAUUCGUAUGAUGAUAAUAUACGUAUUUGUAUGUACAUAAACGAGAAUUUAUCGGUCGGUAAUGUGUAAAUAAGUAAUUAGUGGCGUCUUGACGGGGCCCCGGGCCCCCGUAAACAGUCGAAAGUUCUUAAUUUCUUCAAUAUCUCAUUUCGUUUUGUUAUUGCAAUUUGAUUCUUUGUCUCUCAUUGCGAUUAGAUAUCCUUUAUAAAUGUUGAAAAAAUGCGGGCCACAAAGUUUUUGGACCCAUGUACAUGUUGUGUUCAAAAGUCCUCGCCUAUAAUGUGGGAUUGAUUUCCGAUUUCUCCCAUGUUGUCCCAGCAUGUGGCAAUAUUUGGCACAUUCCAGAUGUAAUACUAGAUGCAUUCCUAAAUGCAAGGCCGUUUUAAACACAUGGACUCACUGGGGGAAGGGGUGCACAAAGGGCCGGCUCACUUGGCAUCAUUGUGGCCGGCUAGUAAGGCAAAAUUUGUCGGCCAUAUGCGUGGUCAGAAUUGUAUGCGUCACUGUCGCACACAUUGGAAUUGUGAUAGGAUUGUACUCACCGCUGUGUUUGUGUAUAUUAGGAACUGCAGCAGGCUAGUAAGGGCCACAGUUCCACAUGAAUCGGCAUCAGUUGCAGCAGUCUAUAAGGGCCACAGUUCUCUGCAGAUUGGCAUUAAUAGCAGUAGGGUUUUCGGUGCAAUUGAAAUUGCUUGUGGUAGGUUUGAAACAGUCCUAUCUUAUACGCAUUUGCAUUAAUCGUGACAGGAUUGUAAAGACAUGCCAUUGUGUGCCAUUGUGCCGUCACAUUAGCACCAGUUAUGACUGAAUGUCAUGCGUCACUGUUACAUACACGUUAGCACCAAUAGUCGCCGGCUGGAAAGAGGCCACAUAUCCACAUGAGUCGUCAUCAGUUGUAGCAUGGUGUAAGAGCCACAGUUUUCUGCAUAUUGUCAUUAAUAUCGGCAGGAUUACAGGCGCAUUGCAUUGAGAUCAAUUGCUGCAACAGUCCUAUCCUAUGAAUUAAUUCUAGCAGGUUUGUAAGGGCCACUGCAUUAUACAUAGUGGCACCAACUCUGGGAGUAUUUUAUGCACCACUGUGUUACACACACACACACACACAUAAGAAGCAAUUACGGCCACAAUCCUCCACACAUCGGCAUCGCUUUUGACAGGUUGCAAGAAACAUAGUUCUCUACAAAUUAAUAUUAAUUUCAGCAAGACUUGCAACAGCCCUAUCUUUUGCGCAUUAGCAUUUGUGGCAGAAUUUCACCCACCGCUGUCCAUCAAUUAUGACAAGCUAGUAAUUGCCACAGUUCUCUACACAUUGGUUGCGGUAGGGUUGUGGGCAUUAUAUGAUAUUGACAUCAGUUUUAGAUUGUUUGUAACCUAUCCUAUGCAAAUGUGAAUUAAUUCUAGCAGGUUUGUCGGCAUGCUAUGCACAUUCGCAUCAAAUGAUGCGGGAAAAAUUGUAUUCGAUGACAUGCACAUAUUCAUAGGCAUCAAUUGUGGUAAACCAGUUAGGGUCAAAGUUCUCUACAUAAUUAGAAUCGAUUGUGGAAGGGUUGCACUCGUCACACAAAUCGACUCCAGUUGUCACAUGCUUGUAAGAGCGAGCUUAAGUGGUACAACAUAAAUGGCUGUGCCACAUCAGCCCAAAACCCCCGGAAUGCCAGGAAGAGAGCCCGUAGUCCCCCAGAAUUUCUGCGAUCAAUUUCCCAGCUGAAACACGUACUGUGUGAAGCCACAUUCCUUGUCACAUUCCCGGAAAUAGCUUUGAAUUACUUAAUUUUUAAACUUUUGGAGUAUAAAAAUGAGUAUAAUUGUAAUAUUUCGCUUUUUAAAUGAUUUUAUAUAGUUAAGGACUAUAAUUAUCUUUCAUGUGUAUUGAAUUCGGAGGCACGAACGAAAAGGAAUAAUUUCUCAGAAAUGUAACUUGAAAUAAUGUUGGAAAUACAUCGAGGGUAUUCUUAUUCCUAUACAUUUUUUGAUAAAUAUUCAAAGUAGAAAUUUAUGUAUAGCUCCAAUAUUUGAUGUAAUAAAAUCUUUCUCUCUCUCUCUUCAAAAAAAA
