# Supplementary material for: Rapid diversification of homothorax expression patterns after gene duplication in spiders
Source: BMC Evol Biol. 2017 Jul 14;17:168. doi: 10.1186/s12862-017-1013-0 (PMC5513375; doi:10.1186/s12862-017-1013-0)
Supplement: Supplementary file 6 — Alignment of Hth1 proteins from four spider species. Abbreviations: Pp, Pholcus phalangioides; Cs, Cupiennius salei; Ag, Acanthoscurria geniculata; Pt, Parasteatoda tepidariorum. Dashes in the alignment denote gaps introduced to improve the alignment. (DOCX 125 kb) [file 12862_2017_1013_MOESM6_ESM.docx]

**Additional file 6. Alignment of Hth1 proteins from four spider species**. Abbreviations: Pp, *Pholcus phalangioides*; Cs, *Cupiennius salei*; Ag, *Acanthoscurria geniculata*; Pt, *Parasteatoda tepidariorum*. Dashes in the alignment denote gaps introduced to improve the alignment.

1 10 20 30 40 50 60

| | | | | | |

Pp-Hth1 MKSEPYPVEHPFATMQYDEGMSHYGSMDGPGSLYDPHGHSRAM--QSLGHAPHMNHTPSM

Cs-Hth1 MKSEPYPLEHSL-AMQYEDGMPHYGAMDGG-PMYDPHGPHRAQMQSLGGHGPHMNHTP-L

Ag-Hth1 ------------------------------------------------------------

Pt-Hth1 MKSEPYPIDHSL-AMQYEDGMPHYGGMDGPTSLYDPHGHRAMQP---LSHAPHMKHTPSM

Pp-Hth1 HQYHGNHVSGVMSNHIMGSVPDVHNKRDKDAIYGHPLFPLLALIFEKCELATCTPREPGI

Cs_Hth1 HQYHGNHVSGVMGNHIMGSVPDVH-KRDKDAIYRHPLFPLLALIFEKCELATCTPREPGI

Ag_Hth1 ------------------------------------MFPLLALIFEKCELATCTPREPGI

Pt-Hth1 HQYHSNHVS--MSNHIMGTVPDVH-KRDKDAIYGHPLFPLLALIFEKCELATCTPREPGI

Pp-Hth1 AGGDVCSSESFNEDITVFAKQIRQEKPYYAPNPELDSLMVQAIQVLRFHLLELEKVHELC

Cs_Hth1 AGGDVCSSESFNEDIAVFAKQIRQEKPYYSPNPELDSLMVQAIQVLRFHLLELEKVHELC

Ag_Hth1 AGGDVCSSESFNEDIAVFAKQIRQEKPYYAPNPELDSLMVQAIQVLRFHLLELEKVHELC

Pt-Hth1 AGGDVCSSESFNEDIAVFAKQIRQEKPYYSPNPELDSLMVQAIQVLRFHLLELEKVHELC

Pp-Hth1 DNFCQRYISCLKGKMPIDLVIDERDSKPGDLGDNNNSSNGGSGNGGGAGGGSGGAGGNGG

Cs_Hth1 DNFCQRYINCLKGKMPIDLVIDERDSKPGDLGDNNNNSSNGGGNGGGAGSGNGG--NPGG

Ag_Hth1 DNFCQRYISRLKGKMPIDLVIDERDSKPGDLGDNNNNSSNGGGGNG---GGNSS--AGGG

Pt-Hth1 DNFCQRYISCLKGKMPIDLVIDERDSKPGDLGEYNNNSSNGGGGAG---GGNSG--AGGG

Pp-Hth1 RGNPDTTGHSSDNSSTPD------QRPPSQSLNSYSTGGEDARSPADSTGTPGPISQQPS

Cs_Hth1 RGNPDTTGHSSDNSSTPDQSFLPYQRPPSQSLNSYSTGPDDARSPAGSTGTPGPISQQPS

Ag_Hth1 RGNPDTTGHSSDNSSTPDQSFVSYQRPPSQSLNSYSTGGEDARSPAGSTGTPGPISQQPS

Pt-Hth1 RGNPDTTGHSSDNSSTPDQSFIPYQRPPSQSLNSYSTGPDDARSPAGSTGTPGPISQQPS

Pp-Hth1 SQMSTDNNSEADVSTYGDASIGSGDGTGEDDDDDRSKKRQKKRGIFPKVATNIMRAWLFQ

Cs_Hth1 SQLSTDNNSEA-----GDASIGSGDGTGEDDDDDRSKKRQKKRGIFPKVATNIMRAWLFQ

Ag_Hth1 SQMSTDNNSEA-----GDASIGSGDGTGEDDDDDRSKKRQKKRGIFPKVATNIMRAWLFQ

Pt-Hth1 SQLSTDNNSEA-----GDASIGSGDGTGEDDDDDRSKKRQKKRGIFPKVATNIMRAWLFQ

Pp-Hth1 HLTHPYPSEDQKKQLAQDTGLTILQVNNWFINARRRIVQPMIDQSNRAGGATAAYGPDGA

Cs_Hth1 HLTHPYPSEDQKKQLAQDTGLTILQVNNWFINARRRIVQPMIDQSNRAGGASAAYGPEGA

Ag_Hth1 HLTHPYPSEDQKKTVGSRYRSHNS------------------------------------

Pt-Hth1 HLTHPYPSEDQ-------------------------------------------------

Pp-Hth1 GMGYMMDGSQQMHIRPPGMQNLSCSDGAMGMGHMGGMGGYSQMSQLRSPVHSQAMLLPGH

Cs_Hth1 GMGYMMDGAQQMHIRPPGMQNLSCSEGAMGMGHMGGMGGYSQMSQLRSPVHSQAMLLPGH

Ag_Hth1 ------------------------------------------------------------

Pt-Hth1 ------------------------------------------------------------

Pp-Hth1 PHAMMMAHGPMGHPGLPPQGSPYDGSGGHIMDIHAS

Cs_Hth1 PHAMMMAHGPMGHPGLPPQGSPYDASGGHIMDIHAS

Ag_Hth1 ------------------------------------

Pt-Hth1 ------------------------------------

Length: 516

Sequences: 4

Identical Sites: 425 (82.4%)

Pairwise % Identity: 88.3%

the percentage of pairwise residues that are identical in the alignment, including gap vs. non gap residues, but excluding gap vs. gap residues
