# Supplementary material for: Rapid diversification of homothorax expression patterns after gene duplication in spiders
Source: BMC Evol Biol. 2017 Jul 14;17:168. doi: 10.1186/s12862-017-1013-0 (PMC5513375; doi:10.1186/s12862-017-1013-0)
Supplement: Supplementary file 7 — Alignment of Hth2 proteins from four spider species. Abbreviations: Pp, Pholcus phalangioides; Cs, Cupiennius salei; Ag, Acanthoscurria geniculata; Pt, Parasteatoda tepidariorum. Dashes in the alignment denote gaps introduced to improve the alignment. (DOCX 126 kb) [file 12862_2017_1013_MOESM7_ESM.docx]

**Additional file 7. Alignment of Hth2 proteins from four spider species.** Abbreviations: Pp, *Pholcus phalangioides*; Cs, *Cupiennius salei*; Ag, *Acanthoscurria geniculata*; Pt, *Parasteatoda tepidariorum*. Dashes in the alignment denote gaps introduced to improve the alignment.

1 10 20 30 40 50 60

| | | | | | |

Pp-Hth2 MQYNEDGIPHPYGVDGGGPPSLYDPHRPMPNLSHHMNHGPSNNLHQYGNSHVNIANHVMG

Pt-Hth2 MQYPEDGMPHYGHGD-GSAGGLYDPHR-QNLMNH---------HGVYHANHVSIANHVMG

Ag-Hth2 ------------------------------------------------------------

Cs-Hth2 MQYGDDGIPHYPAPPQDGPGSLYDAHRHQGIPNH---------HSVYHPNHVAVANHVMG

Pp-Hth2 SMPDVHKRDKDAIYGHPLFPLLALIFEKCELATCTPREPGIPGNDVCSSESFNEDIAVFA

Pt-Hth2 STPDVGKRDKDAIFGHPLFPLLALIFEKCELATCTPREPGIAGGDVCSSESFNEDIACFA

Ag-Hth2 ------------------------------------------------------------

Cs-Hth2 SHPDAHKRDKDAIFGHPLFPLLALIFEKCELATCTPREPGIAGGDVCSSESFNEDITVFA

Pp-Hth2 KQIRQERPYYSPDEELDSIMVQAIQVLRFHLLELEKVHELCDNFCQRYISCLKGKMPIDL

Pt-Hth2 KQIKEERPLYDANPELDSLMVQAIQVLRFHLLELEKVHELCDNFCQRYISCLKGKMPIDL

Ag-Hth2 ------------------------------------------------------------

Cs-Hth2 KQVKEEKPFYVANQELDSIMVQAIQVLRFHLLELEKVHELCDNFCQRYISCLKGKMPIDL

Pp-Hth2 VIEERDTKPELGDTNNNSNGSSYCGGPPCVPRGMMDTSG-HSTDSASTPDQ---------

Pt-Hth2 VIEERDTKPELGDTNNNSNGSSFCGGPPCVSRGMLDTSGGHSTDSGSTPDQGHYEDMSVG

Ag-Hth2 ------------------------------------------------------------

Cs-Hth2 VIDERDTKPELGDTNNNSNGSSFCGGPPCVSRGLLDTSGGHSTDSASTPDQGHYEDIPVG

Pp-Hth2 ------RPPSQSLNYGPVSDDVRSPA-SAGTPCPLSQQPASQQSTDNNSEVGEWDASIGS

Pt-Hth2 NMERLGRPPSQSLNYGSVGDDVRSPTGSTGTPCPLSQQPSSQQSTDNNSEAGD--ASICS

Ag-Hth2 ------RPPSQSLNYSSVGDDVRSPAGSTGTPCPLSQQPSSQQSTDNNSEAGD--ASIGS

Cs-Hth2 SMERLGRPPSQSLNYGSVGDDVRSPNGSSGTPCPMSQQPSSQQSTDNNSEAGD--ASVCS

Pp-Hth2 GEGTGDEDDDDRA-KKNQKKRGIFPKVATNIMRAWLFQHLTHPYPSEDQKKQLAQDTGLT

Pt-Hth2 GEGSGDEDDDERGGKKRQRKRGIFPKVATNILRAWLFQHLTHPYPSEDQKKQLAQDTGLT

Ag-Hth2 GEGSGDDDDDARD-KKRQKTRGIFPKVATNIMRAWLFQHLTHPYPSEDQKK---------

Cs-Hth2 GDGSGDEDDDERG-KKRQKKRGIFPKVATNIMRAWLFQHLTHPYPSEEQKKQLAQDTGLT

Pp-Hth2 ILQVNNWFINARRRIVQPMIDQSNRAGGSIGPPGASYSPESSMGYLMDGVPQMHIRPGLQ

Pt-Hth2 ILQVNNWFINARRRIESLNSGSNCT---HIL-RIKIFNPAGKA-----------------

Ag-Hth2 ------------------------------------------------------------

Cs-Hth2 ILQVNNWFINARRRIVQPMIDQSNRAGGSIA-PGAAYSPESSMGYMIDGSSQMHIRSSSL

Pp-Hth2 G-L---PDSSMGH-MGYSQLRSPVHSQAMLIPGHHAMMMSHPGLPPPPPHGSPYDSSPPN

Pt-Hth2 ------------------------------------------------------------

Ag_Hth2 ------------------------------------------------------------

Cs_Hth2 QNLSCPENMAIGHMAGYSQLRSPVHSQAMLLPGHP-MMMSHPSLP--PPHSSPYDSSPPS

Pp-Hth2 IMDLHSS

Pt-Hth2 -------

Ag_Hth2 -------

Cs_Hth2 IMDLHSS

Length: 487

Sequences: 4

Identical Sites: 330 (67.8%)

Pairwise % Identity: 78.5%

the percentage of pairwise residues that are identical in the alignment, including gap vs. non gap residues, but excluding gap vs. gap residues
